# Adaptive Responses of *Citrus* *grandis* Leaves to Copper Toxicity Revealed by RNA-Seq and Physiology

**DOI:** 10.3390/ijms222112023

**Published:** 2021-11-06

**Authors:** Fenglin Wu, Huiyu Huang, Mingyi Peng, Yinhua Lai, Qianqian Ren, Jiang Zhang, Zengrong Huang, Lintong Yang, Christopher Rensing, Lisong Chen

**Affiliations:** College of Resources and Environment, Fujian Agriculture and Forestry University, Fuzhou 350002, China; 1180807019@fafu.edu.cn (F.W.); 1180807011@fafu.edu.cn (H.H.); 1200807018@fafu.edu.cn (M.P.); 1200807009@fafu.edu.cn (Y.L.); 3200831041@fafu.edu.cn (Q.R.); 2190807006@fafu.edu.cn (J.Z.); huangzengrong@fafu.edu.cn (Z.H.); talstoy@fafu.edu.cn (L.Y.); rensing@fafu.edu.cn (C.R.)

**Keywords:** *Citrus grandis*, copper-toxicity, hormones, leaves, photosynthesis, RNA-Seq

## Abstract

Copper (Cu)-toxic effects on *Citrus grandis* growth and Cu uptake, as well as gene expression and physiological parameters in leaves were investigated. Using RNA-Seq, 715 upregulated and 573 downregulated genes were identified in leaves of *C. grandis* seedlings exposed to Cu-toxicity (LCGSEC). Cu-toxicity altered the expression of 52 genes related to cell wall metabolism, thus impairing cell wall metabolism and lowering leaf growth. Cu-toxicity downregulated the expression of photosynthetic electron transport-related genes, thus reducing CO_2_ assimilation. Some genes involved in thermal energy dissipation, photorespiration, reactive oxygen species scavenging and cell redox homeostasis and some antioxidants (reduced glutathione, phytochelatins, metallothioneins, l-tryptophan and total phenolics) were upregulated in LCGSEC, but they could not protect LCGSEC from oxidative damage. Several adaptive responses might occur in LCGSEC. LCGSEC displayed both enhanced capacities to maintain homeostasis of Cu via reducing Cu uptake by leaves and preventing release of vacuolar Cu into the cytoplasm, and to improve internal detoxification of Cu by accumulating Cu chelators (lignin, reduced glutathione, phytochelatins, metallothioneins, l-tryptophan and total phenolics). The capacities to maintain both energy homeostasis and Ca homeostasis might be upregulated in LCGSEC. Cu-toxicity increased abscisates (auxins) level, thus stimulating stomatal closure and lowering water loss (enhancing water use efficiency and photosynthesis).

## 1. Introduction

Copper (Cu) is required for the proper growth and development of plants, but it is extremely toxic in excess [1,2]. In order to control fruit and leaf fungal diseases, long-term and heavy application of Cu-based fungicides has caused Cu accumulation in soil of *Citrus* orchards. The content of available Cu in the soil increases with *Citrus* planting years. In the old *Citrus* orchards, excessive accumulation of Cu in soil is a common problem limiting *Citrus* production, especially in acidic soil, and is on the rise [3,4,5,6]. The physiological and molecular mechanisms of plant Cu-toxicity and Cu-tolerance have been investigated in some detail. Most studies, however, have focused on phenomena occurring at the roots, because Cu is mainly accumulated in roots exposed to Cu toxicity, and the reduction of root growth has been shown to be usually earlier than that of shoot growth [1,7,8,9]. Less is known about how leaves deal with Cu-toxicity. Growing evidence has shown that Cu-toxicity also influences biosynthesis of photosynthetic pigments, photosynthetic electron transport chain (PETC), CO_2_ assimilation [2,10], production and detoxification of reactive oxygen species (ROS) [11,12], phenol metabolism [13], hormone biosynthesis [14], nitrogen (N) and carbohydrate metabolism [2,15], and cell wall formation [16].

In roots exposed to Cu-toxicity, most of the Cu is bound to the cell wall, thus preventing Cu from entering more sensitive root targets and sensitive shoots, and enhancing Cu-tolerance [17]. Therefore, Cu deposition in the root cell wall is considered as a primary strategy of plant physiology displaying tolerance and detoxification under Cu-toxicity [18,19]. In addition to reducing Cu transport from roots to leaves, different internal detoxification mechanisms of Cu have been developed in leaves, including compartmentation of Cu by import to the vacuole, chelation of Cu by lignin in the cell wall, and intracellular chelation of Cu by Cu chelators [viz., amino acids, reduced glutathione (GSH), phytochelatins (PCs), metallothioneins (MTs), organic acids and phenolics], and induction of Cu-tolerant enzymes [7,9,18,20,21]. It is worth mentioning that Cu binding by the cell wall could impair cell wall metabolism, thus limiting leaf cell growth [22,23]. Phytohormones are key signaling molecules and play an important role in the tolerance to heavy metals (HMs) including Cu [24,25]. Exogenous abscisic acid (ABA) mitigated Cu-toxicity-induced oxidative damage via lowering the accumulation of ROS in *Artemisia annua* leaves [26]. Brassinosteroid (BR) could alleviate Cu-toxic effects on *Raphanus sativus* plants by elevating antioxidant enzyme activities and lowering H_2_O_2_ level [27]. Thus, both the biosynthesis and signaling of some phytohormones might be upregulated in leaves exposed to Cu-toxicity to deal with Cu-toxicity. All these processes should be reflected in gene expression profiles.

RNA-Seq provides a powerful way to elucidate the internal detoxification of Cu in higher plants by monitoring Cu-toxicity-induced alterations of gene expression profiles. Recently, there have been studies investigating Cu-toxic effects on gene expression profiles in plant roots, including melon [28], *Arabidopsis* [29], rice [30] and wheat [31]. There has been evidence showing that Cu-toxic effects on gene expression differ between roots and leaves [18,32,33]. Fu et al. [33] used whole-transcriptome RNA-Seq to investigate the molecular responses of *Citrus junos* roots and leaves to Cu-toxicity. A total of 222 and 5734 mRNAs, five and 164 lncRNAs, 17 and 45 circRNAs, and 130 and 147 miRNAs were differentially expressed in leaves and roots of *C. junos* trees exposed to Cu-toxicity, respectively. By contrast, Cao et al. [32] used RNA-Seq to identify more differentially expressed genes (DEGs) in leaves (1161) than in roots (40) of cucumber seedlings exposed to Cu-toxicity. Wan et al. [18] used qRT-PCR to examine Cu-toxic effects on the expression of genes related to Cu uptake, translocation, homeostasis and detoxification in apple leaves and roots. Cu-toxicity-induced alterations of these genes differed between roots and leaves. However, limited data are available on Cu-toxicity-responsive genes in leaves. Leng et al. [34] and Chen et al. [35] used RNA-Seq to investigate Cu-toxicity-responsive genes in grape leaves. Many genes correlated to ROS detoxification systems (viz., secondary metabolites, antioxidant enzyme and stress-related proteins) were strongly induced. Sudo et al. [36] used DNA microarray to obtained 305 DEGs in leaves of rice plants exposed to Cu-toxicity. A lot of genes involved in the general and the defensive stress response were upregulated, but genes correlated to chlorophyll (Chl) metabolism and photosynthesis were downregulated. In addition, most studies only examined Cu-toxic effects on gene expression profiles in leaves, and did not combine with physiological analysis.

In China, most *Citrus* are commercially planted in acidic soil, which is vulnerable to Cu-toxicity. Here, RNA-Seq was used to investigate Cu-toxicity-responsive genes in *Citrus grandis* leaves. Additionally, we investigated Cu-toxic effects on growth and Cu concentrations in leaves, stems and roots, as well as leaf pigments, gas exchange, relative water content (RWC), hormones, PCs and MTs. The objectives were (a) to test the hypotheses that Cu-toxicity would impair cell wall metabolism, thereby inhibiting leaf growth and that in addition to reducing Cu transport from roots to leaves, internal detoxification mechanisms of Cu (viz., Cu compartmentation, upregulated biosynthesis and signaling of phytohormones) might be involved in leaf Cu-tolerance, and (b) to understand the mechanism underlying Cu-toxicity-induced reduction of photosynthesis at physiological and transcriptional levels.

## 2. Results

### 2.1. Seedling Growth and Cu Level in Roots, Stems and Leaves

**Figure 1:**
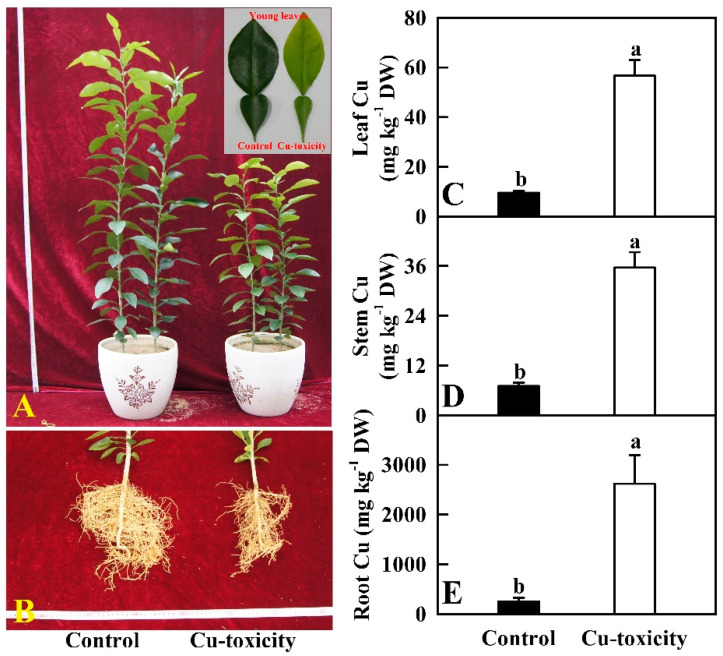
Cu-toxic effects on shoot (**A**) and root (**B**) growth, and mean (SE, *n* = 4) concentrations of Cu in leaves (**C**), stems (**D**) and roots (**E**). Different letters above the bars indicate a significant difference at *p* < 0.05.

‘Shatian’ pummelo [*Citrus grandis*) (L.) Osbeck] is one of the main rootstocks of pummelo. Recent work from our laboratory indicated that ‘Shatian’ pummelo had a higher tolerance to Cu-toxicity and was an ideal material to investigate the adaptive mechanism of Cu-toxicity [2,20]. Herein, 400 μM Cu was chosen as the Cu-toxicity treatment because it led to significant but not too severe alterations of biomass, nutrient uptake, photosynthesis and related parameters in *C. grandis* seedlings [2]. Additionally, we identified more differentially abundant proteins from leaves of 400 μM Cu-treated seedlings than from leaves of 200 or 300 Cu-treated seedlings [20]. As shown in Figure 1, root and shoot growth was inhibited greatly in 400 μM Cu-treated seedlings. Some fibrous roots became dark brown. Young leaf yellowing was observed in some plants. Compared to 0.5 μM Cu treatment (control), the Cu concentrations in leaves, stems and roots of 400 μM Cu-treated seedlings were increased by 494.7%, 408.0% and 929.5%, respectively. Cu concentrations were far higher in roots than in leaves and shoots of 400 μM Cu-treated seedlings. Therefore, most of Cu was accumulated preferentially in roots of 400 μM Cu-treated seedlings.

### 2.2. Gas Exchange, Pigments, RWC, PCs and MTs in Leaves

**Figure 2:**
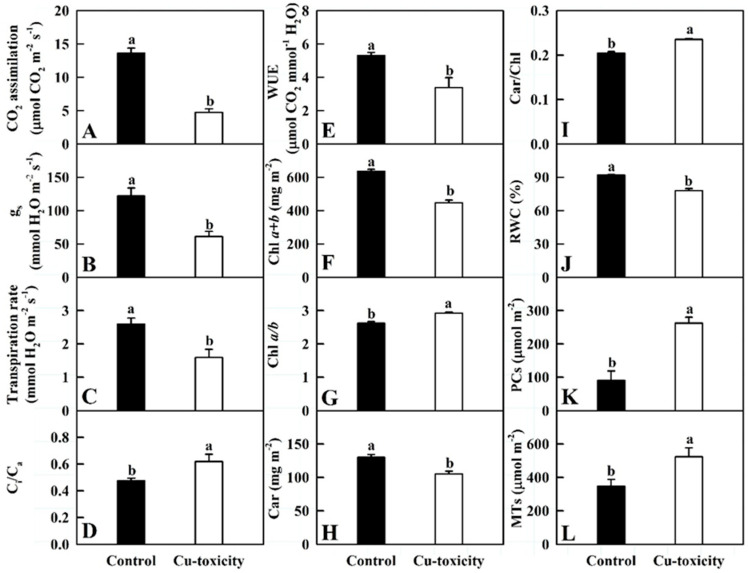
Cu-toxic effects on mean (± SE, *n* = 7 except for 5 for Chl and Car and 4 for RWC, PCs and MTs) CO_2_ assimilation (**A**), stomatal conductance (g_s_, (**B**)), transpiration rate (Tr, (**C**)), ratio of intercellular to ambient CO_2_ concentration (C_i_/C_a_, (**D**)), water use efficiency (WUE, (**E**)), chlorophyll (Chl) *a + b* (**F**), Chl *a*/*b* (**G**), carotenoids (Car, (**H**)), Car/Chl (**I**), relative water content (RWC, (**J**)), phytochelatins (PCs, (**K**)) and metallothioneins (MTs, (**L**)) in leaves. Different letters above the bars indicate a significant difference at *p* < 0.05.

As shown in Figure 2, Cu-toxicity significantly decreased CO_2_ assimilation, stomatal conductance (g_s_), transpiration rate (Tr), water use efficiency (WUE), Chl *a + b*, Car and RWC in leaves by 65.3%, 49.9%, 38.5%, 36.2%, 29.5%, 19.0% and 15.4%, respectively, but it significantly increased the ratio of intercellular to ambient CO_2_ concentration (C_i_/C_a_), Chl *a*/*b*, Car/Chl, PCs and MTs concentrations in leaves by 30.1%, 11.4%, 14.9%, 188.8% and 50.9%, respectively.

### 2.3. RNA-Seq and De Novo Assembly

As shown in Table S1, a total of 47,326,492–5,504,176 raw reads, 46,291,438–53,936,214 clean reads, and 6.94–8.09 G clean bases were generated from six RNA-Seq libraries. Lower error rate (0.02%) and reads related to low quality (0.08–0.13%), N (0.003–0.005%) and adaptor (1.89–3.32%), and higher Q20 (97.11–98.12%), Q30 (91.90–94.42%) and clean reads (96.6–98.0%) suggested that the obtained RNA-Seq data were of high quality, which were suitable for further analysis. Herein, 90.61–92.57% of the clean reads were mapped uniquely to *C. grandis* genome, 44.95% (45.31%) of which were mapped to Read 1 (‘+’ chain) and 45.67% (45.30%) of which were mapped to Read 2 (‘-’ chain), only 2.79%–2.95% of the clean reads were mapped multiply to *C. grandis* genome (Table S2). Similar results have been reported in *C. grandis* leaves [37], roots [38] and fruits [39]. In this study, a total of 21,775 known genes and 2140 novel genes were annotated in *C. grandis* leaves (Tables S3 and S4).

### 2.4. Functional Annotation and Cu-Toxicity-Responsive Genes

**Figure 3:**
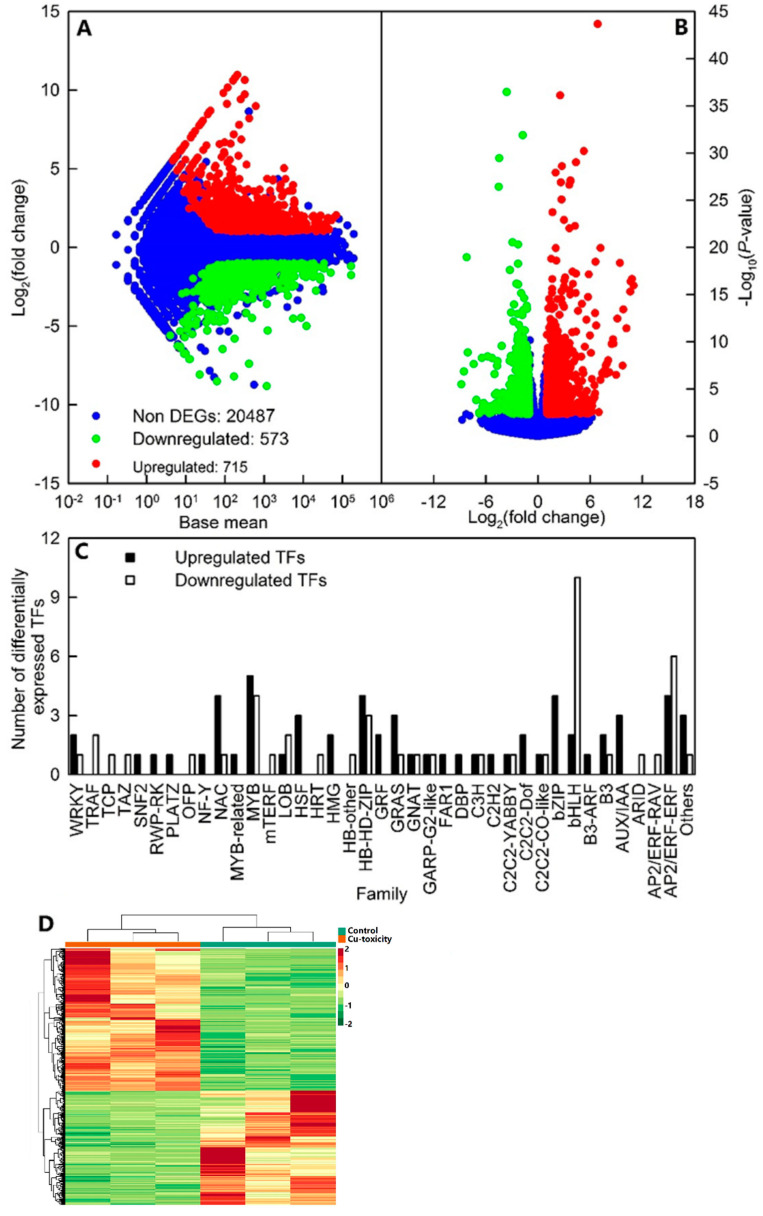
MA map (**A**) and volcano plot (**B**) of differentially expressed genes (DEGs), upregulated and downregulated *transcription factors* (*TFs*, **C**), and cluster analysis of DEGs (**D**) in leaves of *C. grandis* seedlings exposed to Cu-toxicity (LCGSEC).

Here, we identified 573 downregulated and 715 upregulated genes, including 60 upregulated and 45 downregulated *transcription factors* (*TFs*; Figure 3A–C and Tables S5 and S6). Cluster analysis showed that the general expression profiles of DEGs were clustered separately in leaves of seedlings treated with 0.5 and 400 μM Cu, but were clustered together in three biological replicates per treatment (Figure 3D). 

**Figure 4:**
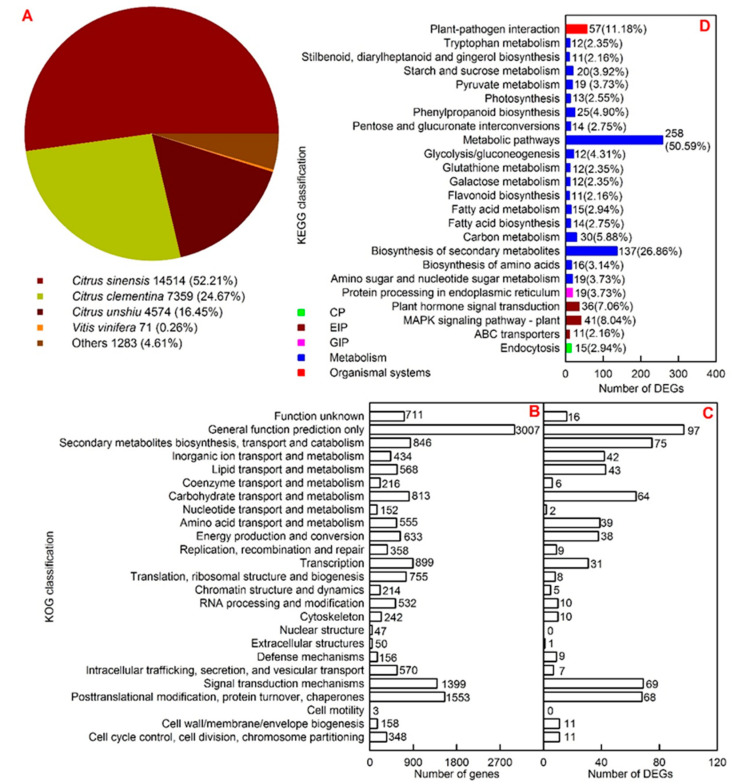
Species distribution of the top Blast hits for *C. grandis* sequences (**A**), KOG classification of all annotated genes in *C. grandis* leaves (**B**) and DEGs in LCGSEC (**C**), and KEGG classification of DEGs in LCGSEC (**D**).

All the assembled high-quality unigenes were first blasted against the National Centre for Biotechnology Information (NCBI) non-redundant protein sequences (NR) database using BLASTX with a cut-off E-value of 10^−5^. Majority of these genes displayed a significant sequence identity to *Citrus sinensis*, *Citrus clementina* and *Citrus unshiu*, which contributed 52.21%, 24.67% and 16.45% of the total assembled genes, respectively (Figure 4A). 

All unigenes and DEGs were submitted to euKaryotic Orthologous Groups (KOG) classification for functional prediction (Figure 4B,C). There were 15,219 annotated genes (671 DEGs) assigned to 25 (23) KOG classifications. For all annotated genes, general functional prediction only (3007) contained the most genes, followed by posttranslational modification, protein turnover, chaperones (1553) and signal transduction mechanisms (1399). For DEGs, KOG classification involving the highest number of DEGs was general functional prediction only (97), followed by signal transduction mechanisms (69) and posttranslational modification (68).

All DEGs were subjected to the Kyoto Encyclopedia of Genes and Genomes (KEGG) database for pathway mapping. A total of 510 DEGs were assigned to 129 KEGG pathways. Metabolic pathways (ko01100) were the KEGG pathway having the highest number of DEGs (258, 50.59%), followed by biosynthesis of secondary metabolites (ko01110, 137, 26.86%) and plant-pathogen interaction (ko04626, 57, 11.18%). Among the 129 KEGG pathways, photosynthesis-antenna proteins (ko00196), fatty acid biosynthesis (ko00061), pyruvate (Pyr) metabolism (ko00620), glycolysis/gluconeogenesis (ko00010), mitogen-activated protein kinases (MAPK) signaling pathway-plant (ko04016), and fatty acid metabolism (ko01212) were significantly enriched at a corrected *p* < 0.05 (Figure 4D and Table S7).

A total of 882 DEGs were assigned to 199 Gene Ontology (GO) terms in cellular component, which of three (viz., photosystem (GO:0009521), photosystem I (PSI; GO:0009522), and PSII (GO:0009523) were significantly enriched with an adjusted *p* < 0.05. A total of 866 DEGs were mapped to 528 GO terms in molecular function, including 17 significantly enriched GO terms. Tetrapyrrole binding (GO:0046906) was the most significantly enriched GO term in molecular function, followed by pigment binding (GO:0031409) and oxidoreductase activity, acting on paired donors, with incorporation or reduction of molecular oxygen, NAD(P)H as one donor, and incorporation of one atom of oxygen (GO:0016709). A total of 763 DEGs were assigned to 1573 GO terms in biological process, including 30 significantly enriched GO terms. The most significantly enriched GO term in biological process was monocarboxylic acid metabolic process (GO:0032787), followed by monocarboxylic acid biosynthetic process (GO:0072330), and photosynthesis, light harvesting in photosystem I (GO:0009768) (Table S8). 

### 2.5. qRT-PCR Analysis

Except for Cg1g015970 and Cg2g038560, Cu-toxicity-induced expression alterations of the other 18 DEGs from RNA-Seq matched well with those from qRT-PCR. There was a significant positive linear correlation between Cu-toxicity-induced alterations of expression levels for the 20 DEGs obtained by qRT-PCR and those obtained by RNA-Seq (Figure S1 and Table S5). Thus, the RNA-Seq results were reliable.

### 2.6. Hormones in Leaves

**Figure 5:**
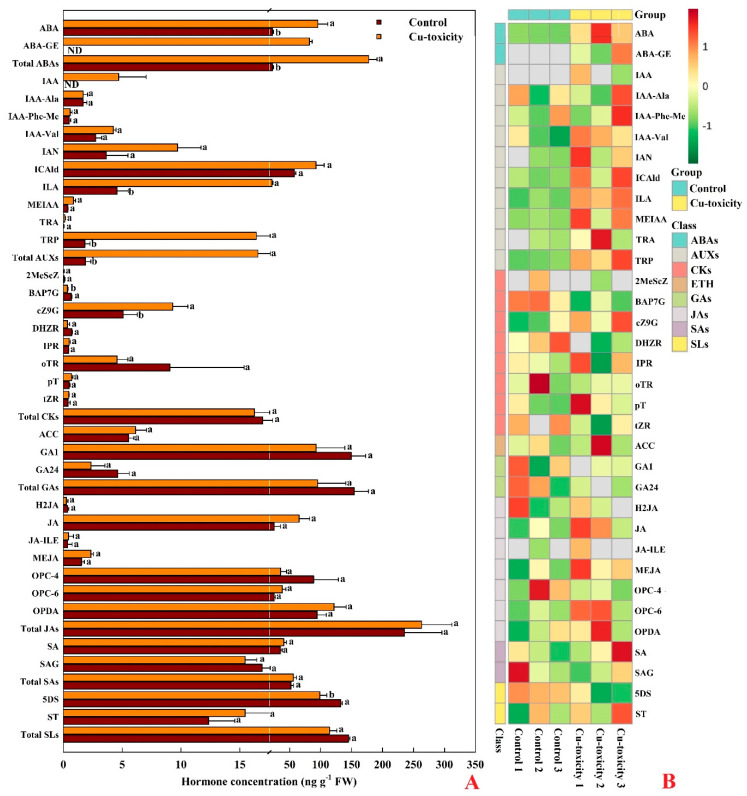
Cu-toxic effects on mean (± SE, *n* = 3) concentrations of hormones in leaves (**A**) and Heatmap of 34 hormones identified in leaves of *C. grandis* seedlings with 0.5 (control) and 400 (Cu-toxicity) μM Cu (**B**). Total ABAs, AUXs, CKs, GAs, JAs, SAs and SLs were the summation of all individual hormone detected for each class. Units for TRP and total AUXs were μg g^−1^ FW. For the same hormone, different letters above the bars indicate a significant difference at *p* < 0.05. ABA-GE, ABA-glucosyl ester; ACC, 1-aminocyclopropanecarboxylic acid; AUXs, auxins; BAP7G, N6-benzyladenine-7-glucoside; CKs, cytokinins; cZ9G, cis-zeatin-9-glucoside; DHZR, dihydrozeatin ribonucleoside; 5DS, 5-deoxystrigol; ETH, ethylene; GA1, gibberellin A1; GA24, gibberellin A24; GAs, gibberellins; H2JA, dihydrojasmonic acid; IAA, indole-3-acetic acid; IAA-Ala, *N*-(3-indolylacetyl)-l-alanine; IAA-Phe-Me, indole-3-acetyl-l-phenylalanne methyl ester; IAA-Val, *N*-(3-indolylacetyl)-l-valine; IAN, 3-indoleacetonitrile; ICAld, indole-3-carboxaldehyde; ILA, indole-3-lactic acid; IPR, N6-isopentenyladenosine; JA, jasmonic acid; JA-ILE, jasmonoyl- L-isoleucine; JAs, jasmonates; MEIAA, methyl indole-3-acetate; MEJA, methyl jasmonate; 2MeScZ, 2-methylthio-cis-zeatin; OPC-4, 3-oxo-2-(2-(Z)-pentenyl) cyclopentane-1-butyric acid; OPC-6, 3-oxo-2-(2-(Z)-pentenyl)cyclopentane-1-hexanoic acid; OPDA, cis(+)-12-oxophytodienoic acid, oTR, ortho-topolin riboside; pT, para-topolin; SA, salicylic acid; SAG, salicylic acid 2-O-β-glucoside; SAs, salicylates; SLs, strigolactones; ST, (±) strigol; TRA, tryptamine; TRP, l-tryptophan; tZR, trans-zeatin riboside.

We detected 34 hormones in leaves, including two ABA and its metabolic products (hereafter referred to as abscisates (ABAs)), namely-ABA and ABA-GE; 10 AUXs, namely-IAA, IAA-Ala, IAA-Phe-Me, IAA-Val, IAN, ICAld, ILA, MEIAA, TRA and TRP; 8 CKs, namely-2MeScZ, BAP7G, cZ9G, DHZR, IPR, oTR, pT and tZR; 1 ETH, namely-ACC; 2 GAs, namely-GA1 and GA24; 7 JAs, namely-H2JA, MEJA, OPC-4, OPC-6, OPDA, JA and JA-ILE; 2 SAs, namely-SA and SAG; and 2 SLs, namely-5DS and ST (Figure 5). Cu-toxicity significantly increased the concentrations of ABA, total ABAs, ILA, TRP, total AUXs and cZ9G by 332.7%, 703.3%, 370.0%, 802.7%, 776.1% and 82.5%, respectively, and significantly decreased the concentrations of BAP7G and 5DS by 49.9% and 25.5%, respectively. Both ABA-GE and IAA were detected only in LCGSEC. The concentrations of the other hormones, total CKs, total GAs, total JAs, total SAs and total SLs in leaves were not significantly altered by Cu-toxicity.

## 3. Discussion

### 3.1. Increased Immobilization of Cu in Roots, and Cu Homeostasis and Detoxification in Leaves

Our results demonstrated that Cu-toxicity increased the accumulation of Cu in *C. grandis* roots (Figure 1), thus limiting Cu to more sensitive shoots and enhancing *C. grandis* Cu-tolerance [17].

To cope with Cu-toxicity, plants have evolved a conserved and complex network of proteins to maintain Cu homeostasis, including Cu transporters, Cu chaperones and Cu-binding proteins [21,40]. Here, we obtained 12 upregulated and 15 downregulated genes related to Cu homeostasis in LCGSEC (Table 1). Transporters responsible for the transport of Cu into the cytoplasm are the high affinity Cu transporter (COPT) family. COPT1 is involved in Cu acquisition and transport into leaves [41]. In *Arabidopsis*, tonoplast COPT5 is important for the export of Cu from the vacuole [42]. Here, we identified one downregulated *COPT5* gene, three downregulated and one upregulated *COPT1* genes in LCGSEC. It is worth mentioning that a total of six *COPT1* genes (Cg8g023340, Cg8g023350, Cg8g023360, Cg8g023370, Cg8g023380 and Cg6g005770) were identified in *C. grandis* leaves (Table S3). Wan et al. [18] reported that Cu-toxicity-induced downregulation of *COPT5* was greater in higher Cu-tolerant HF/Mp than in less Cu-tolerant HF/Mb [‘Hanfu’ (*Malus domestica*) scions grafted on *M. prunifolia* (Mp) and *M. baccata* (Mb), respectively] leaves, and that the expression level of *COPT1* was lower in leaves of HF/Mp than in leaves of HF/Mb when exposed to Cu-toxicity. This might be an adaptive strategy to Cu-toxicity by reducing leaf Cu concentration and preventing the release of vacuolar Cu into the cytoplasm. This agrees with the increased accumulation of Cu in LCGSEC (Figure 1). Cu is transported by the COPT/Ctr-like proteins in its reduced form Cu(I), but most of the bioavailable Cu form in soil is Cu(II). The reduction of Cu (II) to Cu (I) may facilitate the uptake of Cu in roots [40]. Ferric reduction oxidase 2 (FRO2) plays a role in Fe uptake and homeostasis [43]. Additionally, FRO2 can act as a Cu-chelate reductase and facilitate the uptake of Cu [40,44]. FRO7 is involved in Fe uptake by plastids (chloroplasts) [45]. Here, we obtained one upregulated *FRO2* (Cg5g041700) gene, and four upregulated *FRO7* genes (Cg1g023140, Cg2g021360, Cg5g010410 and Cg6g025130) and one downregulated (Cg8g013730) *FRO7* genes in LCGSEC. The Cu-toxicity-induced upregulation of *FRO2* and *FRO7* might be an adaptive strategy by increasing leaf and chloroplast Fe uptake, because excess Cu reduced Fe concentration in *C. grandis* leaves [2]. In addition to the COPT family, zinc (Zn)-regulated transporter (ZRT)- and iron-regulated transporter (IRT)-like proteins (ZIPs) may play a role in Cu uptake [40]. Here, we obtained two downregulated *ZIP* genes (*Zn transporter 4, chloroplastic* and *Zn transporter 1*) in LCGSEC. Wan et al. [18] observed that Cu-toxicity downregulated the expression of *ZIP2* and *ZIP4* in leaves, with a greater degree in HF/Mp than in HF/Mb leaves. The transport of the nicotianamine-metal complexes across plant cell membranes is carried out by the members of the Yellow Stripe-Like (YSL) family [21]. Besides maintaining Fe homeostasis, YSL transporters are involved in distribution and redistribution of Cu [40]. Wan et al. [18] found that *YSL3* was upregulated and downregulated in HF/Mb and HF/Mp leaves, respectively. Cu-toxicity-induced downregulation of *YSL3* and *YSL5* in *C. grandis* leaves might reduce the transport of Cu- nicotianamine from older to younger leaves, thus protecting younger leaves against Cu-toxicity [46].

Cu chaperones can assist Cu intracellular homeostasis by their Cu-chelating ability [47]. Here, we identified one upregulated *Cu chaperone for superoxide dismutase* (*CCS*, Cg5g009340) in LCGSEC. del Pozo et al. [48] reported similar results in roots and shoots of *Arabidopsis* seedlings exposed to Cu-toxicity. Wan et al. [18] reported that Cu-toxicity induced the expression of *CCS* in HF/Mp leaves, but not in HF/Mb leaves. Herein, we obtained three upregulated genes involved Cu ion binding (Cg2g001710, Cg5g007370 and Cg5g009340) and three upregulated *Cu protein* genes (Cg8g018870, Cg2g018560 and Cg3g024840) in LCGSEC, implying that these genes played a role in *C. grandis* Cu-tolerance by binding (cytoplasmic) free Cu ions [40]. However, Cu-toxicity induced the expression of *plastocyanin* (Cg3g024680) and two *cytochrome c oxidase* (Cg9g013180 and CgUng010240) genes in *C. grandis* leaves. This agrees with the report that excess Cu reduced the mRNA transcript levels of *plastocyanin* in *Arabidopsis* leaves [49].

Cu chelators play a role in internal accumulation mechanisms, in which the complexation of Cu can increase Cu immobilization in organelles such as vacuole or cell wall. The present findings and our recent work demonstrated that Cu-toxicity increased the accumulation of PCs, MTs (Figure 2), TRP (Figure 5), GSH, total phenolics and lignin [50] in *C. grandis* leaves, implying that internal accumulation mechanisms played a role in Cu-tolerance of *C. grandis* leaves.

### 3.2. Cu-Toxic Effects on Cell Wall Metabolism in Leaves

As shown in Table S9, 32 downregulated and 20 upregulated genes related to cell wall metabolism were identified in LCGSEC. Wall-associated receptor kinases (WAKs) play a role in cell expansion and in defense against abiotic stress in plants. Hou et al. [51] demonstrated that *WAKL4* expression was induced by excess Cu in *Arabidopsis*, and that an *Arabidopsis WAKL4* T-DNA insertional mutant was hypersensitive to excess Cu. Xia et al. [16] observed that RNAi-mediated *WAK11* knockdown lowered rice Cu-tolerance through enhancing Cu level in cytoplasm of roots and shoots and lowering Cu concentration in the cell wall (pectin and hemicellulose) of roots and shoots due to increased degree of pectin methylesterification, possibly because of decreased activity of pectin methylesterase in roots and shoots. Here, we identified five downregulated and one upregulated pectinesterase genes involved in pectin de-esterification, one downregulated *omega-hydroxypalmitate O-feruloyl transferase* involved in the cell wall pectin biosynthetic process, and two downregulated and two upregulated *WAKs* in LCGSEC, implying that pectin biosynthesis and the degree of pectin methylesterification were downregulated and upregulated in these leaves, respectively, thus decreasing and increasing Cu concentration in the cell wall and cytoplasm, respectively, and hence lowering Cu-tolerance. Xyloglucan is a major hemicellulose component in the cell wall of dicotyledonous plants [52]. Xyloglucan endotransglucosylase/hydrolases (XTHs) were shown to catalyze either the hydrolysis of xyloglucan through xyloglucan endohydrolase (XEH) activity and/or the endotransglycosylation of xyloglucan through xyloglucan endotransglucosylase (XET) activity, thus loosening the cell wall. Zhu et al. [53,54] observed that *xth31* and *xth17 Arabidopsis* mutants had decreased xyloglucan content, slower root elongation, and less aluminum (Al) level in the root tips and cell wall, but higher Al-tolerance than wild-type plants. Here, we obtained two downregulated *XTHs*, indicating that Cu-toxicity might reduce xyloglucan level in LCGSEC, thus decreasing Cu accumulation. This agrees with a report showing that the Cu level was reduced in the cell wall hemicellulose and pectin in *WAK11*-RNAi transgenic rice roots and leaves [16]. To conclude, excess Cu might impair leaf cell wall metabolism, thus inhibiting leaf growth and lowering Cu-tolerance.

### 3.3. Cu-Toxic Effects on Pigment Metabolism, Photosynthesis, and Carbon, Carbohydrate and Energy Metabolisms in Leaves

Cu-toxicity-induced decreases in g_s_ and Tr (Figure 2) agrees with our transcriptome data that Cu-toxicity inhibited the expression of *E3 ubiquitin-protein ligase RZFP34* involved in stomatal opening, but induced the expression of *heat shock 70 kDa protein 1/2/6/8* involved in stomatal closure. However, Cu-toxicity-induced reduction of CO_2_ assimilation was not only explained by reduced g_s_, because C_i_/C_a_ ratio displayed an increasing trend in LCGSEC (Figure 2). The reduction in Chl level (Figure 2) might be due to reduced biosynthesis, as indicated by reduced expression of genes involved in Chl biosynthesis, and increased catabolization, as indicated by increased expression of genes involved in Chl catabolism, while the reduction in Car level (Figure 2) might be due to decreased biosynthesis, as indicated by decreased expression of *geranylgeranyl pyrophosphate synthase* involved in Car biosynthesis. Cu-toxicity-induced inhibition of photosynthesis could not be explained by reduced photosynthetic pigment levels alone, because Cu-toxicity affected CO_2_ assimilation much more than photosynthetic pigments (Figure 2). Here, we isolated 35 downregulated and 12 upregulated genes involved in photosynthesis (ko00195; 12 downregulated and one upregulated genes), photosynthesis-antenna proteins (ko00196; eight downregulated genes), PSI (GO:0009522; 12 downregulated and one upregulated genes), PSII (GO:0009523; 14 downregulated and one upregulated genes), PETC (GO:0009767; three downregulated and one upregulated genes), PSII oxygen evolving complex (OEC, GO:0009654; four downregulated genes), photosynthesis, light reaction (GO:0019684; 12 downregulated and one upregulated genes) and thylakoid (GO:0009579; 29 downregulated and nine upregulated genes). By contrast, we only identified six upregulated genes involved in photosynthesis, dark reaction (GO:0019685) and carbon fixation in photosynthetic organisms (ko00710) (Table S10). Thus, it is reasonable to assume that Cu-toxicity-induced reduction in leaf CO_2_ assimilation was mainly caused by an impaired light reaction, including (*a*) whole PETC (viz., photosynthesis-antenna proteins, light reaction, PSI, PSII, and PSII OEC) and (*b*) thylakoid [2,9,34], rather than by an impaired dark reaction. This is also supported by the report that quite a few of the genes related to PETC were downregulated in leaves of rice plants exposed to Cu-toxicity [36].

We obtained 43 upregulated and 16 downregulated genes involved in carbon, carbohydrate and energy metabolisms, including carbon metabolism (ko01200; 24 upregulated and six downregulated genes), starch and sucrose metabolism (ko00500; 15 upregulated and five downregulated genes), glycolysis/gluconeogenesis (ko00010; 16 upregulated and six downregulated genes), glycolytic process (GO:0006096; eight upregulated genes); Pyr metabolism (ko00620; 17 upregulated and two downregulated genes), citrate cycle [tricarboxylic acid (TCA) cycle] (ko00020; eight upregulated genes), ATP biosynthetic process (GO:0006754; eight upregulated and two downregulated genes), and ATP generation from ADP (GO:0006757; eight upregulated genes) in LCGSEC (Table S11). Thus, these physiological processes might be upregulated in these leaves. Cu-toxicity-induced upregulation of carbon metabolism and starch and sucrose metabolism agrees with increased accumulation of glucose, fructose, sucrose and starch in LCGSEC [2]. The increased accumulation of starch might be due to increased biosynthesis, as indicated by the upregulated expression of two genes encoding 1,4-alpha-glucan branching enzyme and one gene encoding isoamylase involved in starch biosynthesis, and reduced catabolization, as indicated by downregulated expression of two genes (*α-amylase* and *β-amylase*) correlated to starch catabolic process. The higher level of sucrose might be mainly due to elevated biosynthesis, as indicated by enhanced expression of two genes encoding sucrose synthase and one gene encoding sucrose-phosphate synthase, rather than by reduced catabolization, as indicated by elevated expression of one gene encoding β-fructofuranosidase. The elevated levels of glucose and fructose might be associated with increased formation, as indicated by enhanced expression of one *β-fructofuranosidase* gene involved in sucrose degradation, and two *β-glucosidase* and two *glucan endo-1,3-β-glucosidase 1/2/3* genes involved in glucose formation.

Stressed plants often suffer from an energy deficit. Stress tolerance is highly correlated to energy availability in plants. The upregulation of glycolysis in roots of rice and roots and leaves of *Citrus* seedlings exposed to Al has been suggested to be an adaptive mechanism through maintaining basic respiration and meeting an elevated requirement for energy [37,38,55,56]. Mitochondria, the site of respiration and ATP synthesis, have a key role in energy metabolism. Thus, the Cu-toxicity-induced upregulation of genes involved in glycolysis/gluconeogenesis, glycolysis, Pyr metabolism, TCA cycle, ATP biosynthetic process and ATP generation from ADP might be an adaptive strategy for maintaining energy homeostasis and preventing energy shortage.

### 3.4. Cu-Toxic Effects on Thermal Dissipation, ROS Scavenging and Cell Redox Homeostasis in Leaves

More excess excitation energy might exist in LCGSEC due to reduced CO_2_ assimilation (Figure 2). If not rapidly dissipated, the excess excitation energy can potentially stimulate the generation of ROS. Excess excitation energy can be safely removed by xanthophyll cycle-dependent thermal dissipation before it reaches PSII reaction centers (RCs). Photorespiration can also dissipate excess light energy by consuming NADPH and ATP [57]. Cu-toxicity-induced upregulation of *violaxanthin de-epoxidase* involved in xanthophyll cycle and three genes involved in photorespiration (Cg4g009780, Cg6g010200 and Cg4g021720) agrees with the elevated demand for excess light energy dissipation. However, Cu-toxicity inhibited the expression of *catalase* (*CAT*) involved in scavenging H_2_O_2_ produced by photorespiration (Table S12). Because Cu-toxicity stimulated the generation of ROS in *C. grandis* leaves [2], both the expression of some genes correlated to ROS scavenging and the levels of some antioxidants should be altered in LCGSEC. Superoxide dismutase (SOD) can rapidly dismutase superoxide anion into H_2_O_2_ and O_2_. Our findings that *Fe/Mn-SOD* and *Cu/Zn-SOD* (*CSD*) were downregulated and upregulated in LCGSEC, respectively is in agreement with the reports that Fe-SOD abundance was reduced and CSD abundance was elevated in Cu-sufficient *Arabidopsis* leaves [58] and that Cu-toxicity increased CSD abundance, but had no influence on Fe-SOD abundance in *C. grandis* leaves [20]. Plants that suppress *Fe-SOD* and induce *CSD* under Cu-toxicity can keep superoxide anion scavenging and prevent the Cu-toxic effect on photosynthesis by buffering Cu concentration [11,58,59]. Similarly, Cu-toxicity induced the expression of *CCS* in leaves (Table S12), as reported in *Arabidopsis* [59]. CCS has been shown to bind Cu ions and deliver them specifically to CSD, thereby activating CSD [60]. Additionally, both CCS and CSD have been suggested to play a role in Cu homeostasis [18,44]. Ascorbate (ASC) peroxidase (APX) catalyzes the reduction of H_2_O_2_ into H_2_O using ASC as the reducing agent. Davletova et al. [61] reported that cytosolic APX1 played a key role in protecting the chloroplast against photooxidative damage. Here, Cu-toxicity induced the expression of *cytosolic APX2* (Cg6g002810) in leaves (Table S12). Besides protecting plant cells from oxidative damage by quenching reactive molecules by the addition of GSH, glutathione S-transferases (GSTs) play a role in the detoxification of HMs including Cu [62]. Lim et al. [62] found that transgenic *Dianthus superbus* plants overexpressing a tobacco *Tau class GST* (*Nt107*) had higher biomass, CO_2_ assimilation and Cu accumulation than wild type plants when exposed to excess Cu, concluding that transgenic plants enhanced PCs biosynthesis, thereby sequestering and detoxifying excess Cu. Here, we identified one upregulated and seven downregulated *GSTs* in LCGSEC (Table S12). This agrees with our report that LCGSEC had increased accumulation of GSH [50]. However, Leng et al. [34] identified 27 upregulated and three downregulated *GSTs* in grape leaves exposed to Cu-toxicity. Peroxidases (PODs) can reduce H_2_O_2_ to H_2_O using a wide variety of substrates as electron donor. Here, we obtained two upregulated and five downregulated *PODs* in LCGSEC. However, only seven upregulated *PODs* were observed in grape leaves exposed to Cu-toxicity [34]. Additionally, we obtained three downregulated and five upregulated genes related to cell redox homeostasis (GO:0045454) in LCGSEC. In conclusion, some genes correlated to thermal dissipation, photorespiration, ROS scavenging and cell redox homeostasis (Table S12) and the levels of some antioxidants such as PCs, MTs, TRP (Figure 2 and Figure 5), GSH and total phenolics [50] were upregulated in LCGSEC, but they could not sufficiently protect these leaves from oxidative damage, because MDA level [50] and electrolyte leakage [2] were increased in LCGSEC.

### 3.5. Cu-Toxic Effects on Calcium Signaling and MAPK Signaling in Leaves

Calcium (Ca) signaling and MAPK signaling are the major signaling networks involved in the toxicity of HMs including Cu [24]. We obtained 29 downregulated and 11 upregulated genes involved in Ca homeostasis, including Ca ion transmembrane transporter activity (GO:0015085; five downregulated and four upregulated genes), Ca-mediated signaling (GO:0019722; one upregulated and one downregulated genes), CaM binding (GO:0005516; 12 downregulated and five upregulated genes), CaM-dependent protein kinase activity (GO:0004683; one upregulated gene), Ca ion binding (GO:0005509; 14 downregulated and three upregulated genes), and Ca channel activity (GO:0005262; two upregulated genes) in LCGSEC (Table S13). In plants, various Ca^2+^-binding proteins can act as Ca^2+^-sensors to monitor the alterations of cytosolic Ca^2+^ concentration ([Ca^2+^]_cyt_), including calmodulins (CaMs), calcineurin B-like proteins (CBLs), CaM like proteins (CMLs), and Ca^2+^-dependent protein kinases (CDPKs) [24]. CaMs work through binding to and regulating the activities of diverse downstream target proteins called “CaM-binding proteins” (CaMBPs), which provide another level of specificity for Ca signaling [63]. Ca^2+^ transport molecules, Ca^2+^ buffers and Ca^2+^ sensors are involved in the maintenance of Ca^2+^ homeostasis [64]. Here, we identified three downregulated [*Ca-transporting ATPase 9, plasma membrane-type* (*ACA9*, Cg5g012700) and *ACA12* (Cg2g020060 and Cg5g034830)] and one upregulated [*ACA12* (Cg3g010620)] *AUTOINHIBITED Ca^2+^-ATPase* (*ACA*) genes involved in the translocation of Ca^2+^ from the cytosol out of the cell or into the organelle, one upregulated *Ca-transporting ATPase 4, endoplasmic reticulum (ER)-type* (*ECA4*, Cg6g003500) involved in the translocation of Ca^2+^ from the cytosol into an endomembrane compartment, two downregulated *vacuolar cation/proton exchanger 3 (CAX3)* genes (Cg6g006900 and Cg8g024060) involved in the translocation of Ca^2+^ into the vacuole; one upregulated *Ca uniporter protein 2, mitochondrial* (Cg3g022310) involved in the uptake of mitochondrial Ca, and one upregulated *glutamate receptor 2.8* (GLR2.8, Cg4g022490), a non-selective cation channel involved in the regulation of Ca^2+^ influx into cell in LCGSEC (Table S13), indicating that Ca^2+^ influx into the cytosol and Ca^2+^ influx into endomembrane compartment (ER and mitochondrium) might be enhanced, but Ca^2+^ efflux out of cytosol into the cell exterior and sequestration into vacuole might be downregulated in these leaves, thereby maintaining Ca^2+^ homeostasis in the cytosol and the endomembrane compartment, because excess Cu reduced Ca level in *C. grandis* leaves [2] and that Cg3g010620 (*ACA12*) might be involved in the translocation of Ca^2+^ from the cytosol into the organelle. Here, we obtained 14 downregulated and three upregulated genes involved in Ca^2+^ binding in LCGSEC, indicating that the amount of Ca^2+^ bound to Ca^2+^ buffering proteins might be reduced in these leaves, thus contributing to Ca^2+^ homeostasis in the cytosol. Additionally, most of DEGs [12 downregulated (Cg2g020060, Cg3g013020, Cg3g012090, Cg8g018400, Cg2g014880, Cg8g018410, Cg5g018340, Cg8g018470, Cg5g006140, Cg5g034830, Cg4g002250 and Cg5g012700) and five upregulated (Cg9g029620, Cg9g027140, Cg2g043490, Cg3g010620 and Cg8g010170) genes] involved in CaM binding were downregulated in LCGSEC.

We obtained one upregulated *GLR2.8* (Cg4g022490) and one downregulated *protein RALF-like 32* (*RALFL32*; Cg9g014450) related to Ca-mediated signaling, and one upregulated [*Ca/CaM-dependent protein kinase (CaM kinase) II* (*CCaMKII*, Cg8g010170)] and six downregulated [four *CMLs* (Cg1g003570, Cg2g011280, Cg3g017200 and Cg6g011100), *CaM* (Cg3g021480) and *CBL* (Cg5g018200)] genes encoding Ca sensors in LCGSEC (Table S13). A deletion of *AtGLR3.5* caused premature senescence and a decrease in Chl level in *Arabidopsis* leaves [65]. A deletion of *AtGLR3.4* led to decreased photosynthetic yield of PSII and non-photochemical quenching [66]. ZmCCaMK is considered to play a key role in ABA- and BR-induced antioxidant protection in maize leaves [67,68]. Rapid alkalinization factor (RALF) led to rapid alkalization of the cell wall by mediating a transient elevation of [Ca^2+^]_cyt_, thus inhibiting cell growth in tissue culture [69]. Transgenic *Arabidopsis* plants overexpressing *RALF22* or *RALF23* displayed decreased growth and less tolerance to salt stress [70]. Thus, Cu-toxicity-induced upregulation of *GLR2.8* and *CCaMKII* and downregulation of *RALFL32* might contribute to *C. grandis* Cu-tolerance. Except for *CCaMKII*, all the other six Ca sensor genes were downregulated in LCGSEC, probably contributing to Ca^2+^ homeostasis, because all the six Ca sensors were Ca^2+^ binding proteins.

We identified 29 downregulated and 12 upregulated genes involved in the MAPK signaling pathway-plant (ko04016) in LCGSEC (Table S14). MAPK-signaling cascade includes three protein kinases [MAPKs, MAPK kinases (MAPKKs) and MAPKK kinases (MAPKKKs)] acting in a sequential manner to activate different downstream targets. MAPKs can be stimulated by specific metal ligands or indirectly by ROS produced due to metal stress [24]. Yeh et al. [71] reported that Cu^2+^ induced MAPK activation by distinct ROS generating systems in rice roots. Additionally, ABA induced a rapid and transient MAPK activation in pea leaves [72]. Thus, Cu-toxicity might increase ABA accumulation (Figure 5) and ROS generation [2], thus inducing *MAPKK4/5* (*MKK4/5*) expression in *C. grandis* leaves. This agrees with a report that two novel rice *MAPK* genes (*OsMSRMK3* and *OsWJUMK1*) in rice leaves were induced by Cu-toxicity, ABA and H_2_O_2_ [73]. These results demonstrated the involvement of MAPKs in mediating Cu-toxicity. Protein phosphatases 2C (PP2Cs) are the negative regulators of stress-induced receptor kinase signaling, MAPK pathways and ABA signaling [74]. Here, we identified five upregulated genes encoding protein phosphatase 2C (PP2C) in LCGSEC (Table S14). Overexpression of an ABA, salt and drought inducible rice *PP2C* gene, *OsPP108* conferred *Arabidopsis* tolerance to drought, mannitol, salt stress and ABA insensitivity [75]. Liu et al. [76] indicated that *A. thaliana* protein phosphatase 2C G Group 1 positively regulated salt stress in an ABA-dependent manner. Therefore, the upregulation of *PP2Cs* might be an adaptive response to Cu-toxicity. To conclude, our findings demonstrated the involvement of the MAPK signaling pathway in Cu-tolerance and toxicity.

### 3.6. Cu-Toxic Effects on Biosynthesis and Signaling of Phytohormones in Leaves

As shown in Tables S15–S17, we identified 24 upregulated and 12 downregulated genes involved in plant hormone signal transduction (ko04075), 37 upregulated and 28 downregulated genes involved in auxin (AUX)-, GA-, ETH-, BR-, ABA-, SA-, CK- and JA-mediated or activated signaling pathways, 22 upregulated and 15 downregulated genes involved in ABA, CK, AUX, BR, SA, JA and GA metabolism in LCGSEC. Cu-toxicity increased the concentrations of ABA, ILA, TRP, and cZ9G, decreased the concentrations of BAP7G and 5DS, but did not affect the concentrations of the other 28 hormones in leaves (Figure 5). These results indicated the involvement of hormone signaling in Cu-tolerance of *C. grandis* leaves.

Ouzounidou and Ilias [77] demonstrated that IAA alleviated Cu-toxic effects on sunflower seedlings by improving photosynthesis and WUE. Leng et al. [34] reported that all *AUX response factors* (*ARFs*) were inhibited and most genes encoding IAA synthase and AUX/IAA proteins were induced in grape leaves exposed to Cu-toxicity, suggesting that the IAA regulated genes might positively regulate grape development and Cu-tolerance. Here, we obtained four upregulated and two downregulated genes correlated to AUX signal transduction, 11 upregulated and eight downregulated genes correlated to AUX-activated signaling pathway, four upregulated and one downregulated genes correlated to AUX biosynthetic process, and seven upregulated and three downregulated genes correlated to AUX transport in LCGSEC. Additionally, Cu-toxicity increased the levels of IAA, TRP and total AUXs in leaves (Figure 5). These results suggested that AUX biosynthesis, levels and signal signaling might be upregulated in LCGSEC, which might enhance Cu-tolerance of *C. grandis* leaves via promoting CO_2_ assimilation and WUE.

We detected six upregulated [five *PP2Cs* and one *ABA responsive element binding factor* (*ABF*)] and two downregulated (two *ABA receptor PYL4*) and 11 upregulated and 8 downregulated genes involved in ABA signal transduction and ABA-mediated signaling pathway, respectively in LCGSEC, implying that ABA signaling might be upregulated in these leaves. However, one upregulated and four downregulated genes correlated to the ABA biosynthetic process were identified in LCGSEC. The downregulation of ABA biosynthesis-related genes caused by Cu-toxicity might be due to feedback inhibition caused by large increase of ABA level in these leaves [78]. This agrees with the reports that both the level of ABA and the expression of genes [*CsPP2C5*, *CsABI1*, *Cucumis sativus SNF1-related kinase 2.3* (*CsSnRK2.3*), *CsSnRK2.4* and *most CsPYLs*] involved in ABA signaling were upregulated in cucumber seeds exposed to Cu-toxicity [79], and that the level of ABA was increased in leaves of sunflower seedlings exposed to Cu-toxicity [80]. ABA is well-known for its ability to stimulate stomatal closure. Cu-toxicity-induced increase in ABA level might stimulate stomatal closure, thus reducing transpiration water loss, because g_s_, Tr and RWC were reduced in LCGSEC (Figure 2). ABA has been suggested to have a positive and synergistic relationship with the biosynthesis of GSH and PCs [81]. Cu-toxicity-induced increase of ABA concentration might improve the biosynthesis of GSH and PCs, as indicated by increased accumulation of GSH [50] and PCs (Figure 5) in LCGSEC. Zehra et al. [26] reported that ABA alleviated Cu-toxicity-induced oxidative damage in *A. annua* leaves by reducing ROS production. The increased accumulation of ABA is also in agreement with the increased requirement for ROS scavenging [2].

One upregulated *histidine-containing phosphotransfer peotein 4* (*AHP4*) and one downregulated *two-component response regulator ARR-B family* (*ARR-B*) involved in CK signal transduction, two upregulated genes (Cg1g013900 and Cg5g001740) involved in CK-activated signaling pathway, and one upregulated *CK riboside 5′-monophosphate phosphoribohydrolase LOG7* involved in the conversion from inactive CK nucleotides to the biologically active free-base forms [82] were identified in LCGSEC (Table S17). Additionally, Cu-toxicity increased cZ9G levels, and decreased BAP7G levels, but did not significantly alter the levels of total CKs and other CKs in leaves (Figure 5). Thus, both the levels of the active free-base form CKs and CK signaling might be upregulated in these leaves. Thomas et al. [83] reported that *isopentenyltransferase* (*IPT*)-induced CKs in transgenic tobacco enhanced Cu-tolerance and decreased Cu-toxicity-induced lipid peroxidation in leaves, which was explained by an upregulated expression of a *MT-like* gene (*MT-L2*) in leaves. Cu-toxicity might increase the levels of active CKs, thus enhancing the level of MTs and Cu-tolerance of *C. grandis* leaves.

## 4. Materials and Methods

### 4.1. Plant Culture and Cu Treatments

Plant culture and Cu treatments were carried out as described by Li et al. [2] and Huang et al. [50]. Briefly, ~13-week-old ‘Shatian’ pummelo (*Citrus grandis* (L.) Osbeck) seedlings grown in 6 L pots (two plants pot^−1^) containing sand in a greenhouse with natural photoperiod at Fujian Agriculture and Forestry University, Fuzhou (26°5′ N, 119°14′ E) with annual average sunshine hours, temperature and relative humidity of ~1600 h, 20 °C and 76%, respectively [84], were irrigated 6 times weekly for 6 months with freshly prepared nutrition solution at a Cu level of 0.5 or 400 μM from CuCl_2_ until there was nutrient solution leaking out of the small hole at the bottom of the pot (~500 mL). The pH of the nutrient solutions was adjusted to 4.8 with HCl to prevent Cu precipitation. Twenty pots (a total of 40 plants) in each treatment were arranged at random. Thereafter, the most recent, fully expanded mature leaves at ~7 weeks of age were selected for all analyses. After leaf gas exchange measurements, leaves without petioles, winged leaves and midribs and leaf discs of 6-mm-diameter were harvested from the same plants used for gas exchange measurements at sunny noon, frozen in liquid N_2_, and then stored at –80 °C until RNA and metabolite extraction. The unsampled seedlings were selected for the measurements of Cu in leaves, stems and roots, and RWC in leaves.

### 4.2. Cu Concentration in Leaves, Stems and Roots

The fully expanded mature leaves without petioles, midribs and winged leaves, the middle sections of stems and the fibrous roots were taken for subsequent analysis [85]. Leaf, stem and root Cu concentration was determined with a NexION 300X Inductively Coupled Plasma Mass Spectrometer (ICP-MS, PerkinElmer, Shelton, CT, USA) after 0.2 g of samples were digested in 5:1 (v:v) of HNO_3_:HClO_4_ [2].

### 4.3. Gas Exchange, Pigments, RWC, PCs and MTs in Leaves

Leaf gas exchange measurements were performed with a CIRAS-2 portable photosynthesis system (PP systems, Herts, UK) at an ambient CO_2_ concentration (~410 µmol mol^−1^) and a controlled light intensity of ~1000 µmol m^−2^ s^−1^ between 9:30 and 10:30 a.m. on a sunny day [2].

Leaf Chl *a*, Chl *b* and carotenoids (Car) concentrations were measured after being extracted with 80% (v:v) acetone [86].

Leaf RWC were assayed using weighing method [87].

Leaf GSH and total non-protein thiols (TNP-SH) were extracted and assayed according to Garg and Kaur [88]. Four leaf discs (0.2826 cm^2^ in size) were extracted with 2 mL of ice-cold 5% (w:v) sulphosalicylic acid. For the GSH assay, 0.5 mL of supernatant was mixed with 0.6 mL of 100 mM (pH 7.0) phosphate buffer and 40 µL of 1 mM 5,5-dithiobis-2-nitrobenzoic acid (DTNB). After 2 min, the absorbance was read at 412 nm. For the TNP-SH assay, 100 μL of supernatant was mixed with 0.5 mL of 0.1 M phosphate buffer (pH 7.0) containing 0.5 mM EDTA and 0.5 mL of 1 mM DTNB. After 10 min, the absorbance was read at 412 nm. The difference between TNP-SH and GSH was considered to represent PCs.

Leaf MTs was extracted and measured according to Malik et al. [89]. Four leaf discs (0.2826 cm^2^ in size) were homogenized in 1.6 mL of ice-cold solution containing 0.5 M sucrose, 20 mM Tris-HCl buffer, pH 8.6 and 0.01% β-mercaptoethanol. After being centrifuged at 30,000× *g* for 20 min. 1 mL of supernatant was mixed with 1 mL of cold ethanol and 80 μL of chloroform; the mixtures were then centrifuged at 6000× *g* for 10 min at 4 ºC. The collected supernatant was mixed with 1 mg RNA and 40 μL of 37% HCl and subsequently with 3 mL of cold ethanol. The sample was maintained at –20 °C for 1 h, then centrifuged at 6000× *g* for 10 min. The MTs containing pellet was washed with 87% ethanol and was re-suspended in 150 μL of 0.25 M NaCl and 150 μL of 1 M HCl containing 4 mM EDTA. A volume of 4.2 mL NaCl (2 M) containing 0.43 mM DTNB buffered with 0.2 M Na-phosphate (pH 8) was added to the sample at room temperature. The sample was finally centrifuged at 3000× *g* for 5 min, and the supernatant was measured as absorbance at 412 nm.

### 4.4. Leaf RNA Extraction and RNA-Seq

Total RNA was extracted from ~200 mg of frozen leaves mixed equally from four plants (one plant pot^−1^) using Recalcitrant Plant Total RNA Extraction Kit (Bioteke Corporation, Beijing, China). There were three biological replicates per treatment. A total of six sequencing libraries were constructed according to Guo et al. [37,38] and sequenced on Illumina HiSeq platform (Illumina Inc., San Diego, CA, USA) at Wuhan MetWare Biotechnology Co., Ltd. (www.metware.cn, accessed on 1 March 2021). The raw transcriptome data have been deposited in NCBI SRA database (accession number: PRJNA702620).

### 4.5. RNA-Seq Analysis

The raw sequencing reads were filtered using fastp v0.7.0 [90]. High-quality clean reads obtained after filtering were mapped to the genome of pummelo downloaded from the genome website (http://citrus.hzau.edu.cn/orange/download/index.php, accessed on 1 January 2021) directly using HISAT2. The mapped reads of each sample were assembled using StringTie v1.3.4b [91]. The expression level of each gene was given as fragment per kilobase of transcript per million mapped reads (FPKM). Differential expression analysis between two samples was carried out by DESeq2 software using the counts from featureCounts [92]. Then, we used Benjamini and Hochberg’s method to adjust the *p*-valve and obtain the false discovery rate (FDR). Screening criteria for DEGs were |log_2_(fold change)| ≥ 1 and FDR < 0.05. Gene functions were annotated according to Swiss-Prot, TrEMBL, KEGG, NR, GO, KOG and Protein family (Pfam) [93].

### 4.6. qRT-PCR Validation

Twenty DEGs were randomly selected for qRT-PCR validation. Total RNA was extracted from leaves according to the method described above. There were 3 replicates per treatment. The sequences of the Forward and Reverse primers designed using Primier version 5.0 (Premier Biosoft International, CA, USA) were listed in Table S18. qRT-PCR was run in 2 technical replicates. *U4/U6 small nuclear ribonucleoprotein PRP31* (*PRPF31*; Cg7g019550) and *actin* (Cg1g026080) were selected as internal standards [37].

### 4.7. Extraction and Measurements of Hormones in Leaves

Approximately 50 mg frozen leaves were ground into powder in liquid N_2_, and extracted with 1 mL of methanol:water:formic acid (15:4:1, v:v:v). After centrifugation, the extracts were collected, evaporated to dryness in N_2_ flow, reconstituted in 100 μL of 80% methanol (*v*/*v*), and filtered through a 0.22 μm filter for further liquid chromatograph-mass spectrometer (LC-MS) analysis. Hormones were assayed by MetWare (http://www.metware.cn/, accessed on 1 March 2021) based on the AB Sciex QTRAP 6500 liquid chromatography-tandem mass spectrometry (LC-MS/MS) platform [94].

### 4.8. Statistical Analysis

Results represented the mean ± SE (*n* = 3–5). Comparison between two treatment means was carried out in SigmaPlot 10 (Systat Software, Inc., San Jose, CA, USA, http://systasoftware.com, accessed on 1 May 2021) using a one-tailed *t*-test at *p* < 0.05. For Hierarchical Cluster Analysis (HCA), the data were normalized and analyzed by R software (https://www.r-project.org/, accessed on 1 May 2021).

## 5. Conclusions

**Figure 6:**
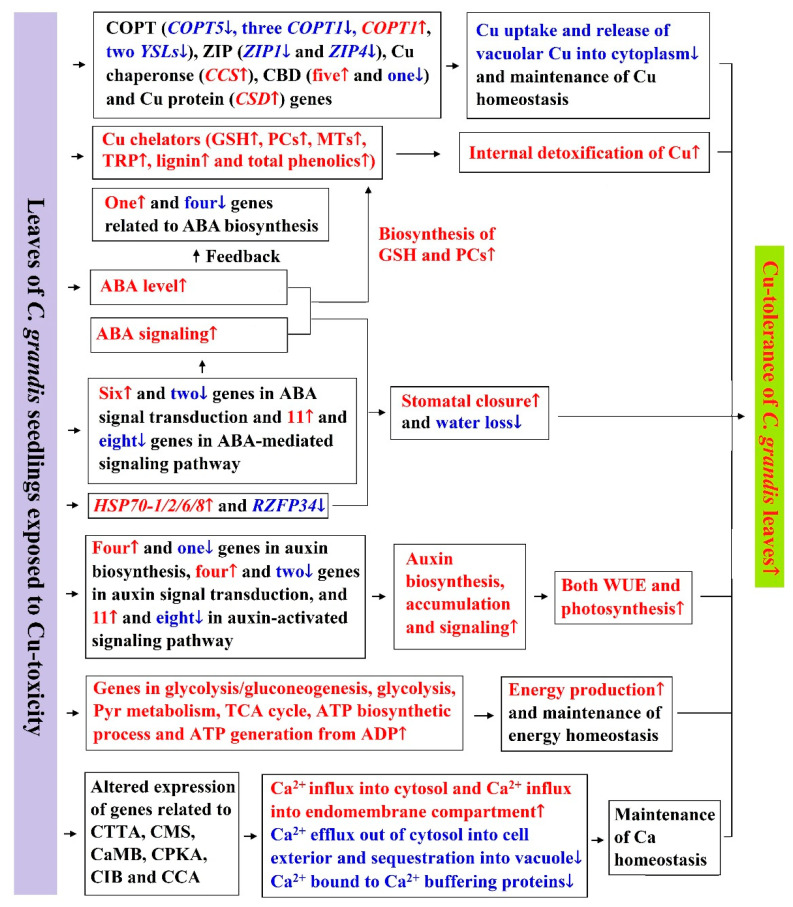
A potential model for the adaptive responses of *C. grandis* leaves to Cu-toxicity. CaMB, CaM binding; CCA, Ca channel activity; CIB, Ca ion binding; CMS, Ca-mediated signaling; CPKA, CaM-dependent protein kinase activity; CTTA, Ca ion transmembrane transporter activity; HSP70-1/2/6/8, heat shock 70kDa protein 1/2/6/8; Red, upregulation; ↑,upregulation; Blue, downregulation; ↓, downregulation.

Thirty-two upregulated and 20 downregulated genes related to cell wall metabolism were identified in LCGSEC, implying that Cu-toxicity might impair cell wall metabolism, thus reducing leaf growth and lowering Cu-tolerance. Cu-toxicity decreased and increased the expression of genes involved in Chl biosynthesis and degradation, respectively, thus reducing Chl concentration, while Cu-toxicity-induced reduction in Car concentration might be mainly due to decreased biosynthesis. The reduction in leaf CO_2_ assimilation caused by Cu-toxicity might be due to impaired PETC, as indicated by the downregulated expression of related genes. Although some genes related to thermal dissipation, photorespiration, ROS scavenging and cell redox homeostasis and some antioxidants were upregulated in LCGSEC, but antioxidant systems as a whole could not protect LCGSEC from oxidative damage. In addition to reducing Cu transport from roots to shoots, several adaptive responses might occur in LCGSEC. A model for the adaptive responses of *C. grandis* leaves to Cu-toxicity was proposed through the integration of the present findings and available data in the previous literatures (Figure 6). LCGSEC displayed enhanced capacities to maintain homeostasis of Cu via reducing Cu uptake by leaves and preventing vacuolar Cu into the cytoplasm, as indicated by altered expression of genes encoding Cu transporters, Cu chaperones and Cu-binding proteins and to improve internal detoxification of Cu, as indicated by increased accumulation of selected Cu chelators. LCGSEC displayed increased capacities to maintain both energy homeostasis by upregulating the expression of genes involved in energy (ATP) production and Ca homeostasis by altering the expression of related genes. Cu-toxicity increased the concentrations of ABAs (AUXs), thus stimulating stomatal closure and reducing water loss (improving WUE and photosynthesis).

## Figures and Tables

**Table 1:** DEGs related to Cu homeostasis in LCGSEC.

Accession No.	KEGG	Swiss-Prot	Log_2_(FC)
Cu ion transmembrane transporter activity (GO:0005375)			
Cg4g018610	Solute carrier family 31 (copper transporter), member 1	Copper transporter 5; AtCOPT5	−1.317
Cg8g023350	Solute carrier family 31 (copper transporter), member 1	Copper transporter 1; AtCOPT1	−3.606
Cg8g023360	Solute carrier family 31 (copper transporter), member 1	Copper transporter 1; AtCOPT1	−1.076
Cg8g023380	Solute carrier family 31 (copper transporter), member 1	Copper transporter 1; AtCOPT1	−8.212
Cg6g005770	Solute carrier family 31 (copper transporter), member 1	Copper transporter 1; AtCOPT1	3.407
Yellow Stripe-Like (YSL) family			
Cg5g018670	Fanconi-associated nuclease 1 [EC:3.1.21.- 3.1.4.1]	Metal-nicotianamine transporter YSL3; Protein YELLOW STRIPE LIKE 3; AtYSL3	−1.609
Cg5g020560	Fanconi-associated nuclease 1 [EC:3.1.21.- 3.1.4.1]	Probable metal-nicotianamine transporter YSL5; Protein YELLOW STRIPE LIKE 5; AtYSL5	−1.066
Cu ion binding (GO:0005507) and/or Cu proteins			
Cg1g028930	l-ascorbate oxidase [EC:1.10.3.3]	l-ascorbate oxidase	−3.067
Cg2g001710	Enoyl-[acyl-carrier protein] reductase I [EC:1.3.1.9 1.3.1.10]	Enoyl-[acyl-carrier-protein] reductase [NADH], chloroplastic	1.679
Cg2g018560	Iron transport multicopper oxidase	l-ascorbate oxidase homolog	1.069
Cg3g024840	Iron transport multicopper oxidase	l-ascorbate oxidase homolog	1.455
Cg3g024680	Plastocyanin	Plastocyanin, chloroplastic	−1.448
Cg5g007370	Glutamate dehydrogenase (NAD(P)^+^) [EC:1.4.1.3]	Glutamate dehydrogenase 2	1.745
Cg5g009340	Copper chaperone for superoxide dismutase	Copper chaperone for superoxide dismutase, chloroplastic/cytosolic; AtCCS	1.588
Cg7g012360	Glutathione S-transferase [EC:2.5.1.18]	Glutathione S-transferase F9	−1.318
Cg8g018870	Superoxide dismutase, Cu-Zn family [EC:1.15.1.1]	Superoxide dismutase [Cu-Zn], chloroplastic	1.344
Cg9g013180	Cytochrome c oxidase subunit 3	Cytochrome c oxidase subunit 3	−1.324
CgUng010240	Cytochrome c oxidase subunit 2	Uncharacterized mitochondrial protein AtMg00530	−1.581
Cu chaperones			
Cg5g009340	Copper chaperone for superoxide dismutase	Copper chaperone for superoxide dismutase, chloroplastic/cytosolic; AtCCS	1.588
Others			
Cg3g000750	Cd^2+^/Zn^2+^-exporting ATPase [EC:3.6.3.3 3.6.3.5]	Cadmium/zinc-transporting ATPase HMA2	−1.403
Cg4g006740	Solute carrier family 39 (zinc transporter), member 1/2/3	Zinc transporter 4, chloroplastic; ZRT/IRT-like protein 4	−1.718
Cg8g022750	Solute carrier family 39 (zinc transporter), member 1/2/3	Zinc transporter 1; ZRT/IRT-like protein 1; OsZIP1	−2.937
Cg5g041700	Ferric-chelate reductase [EC:1.16.1.7]	Ferric reduction oxidase 2; AtFRO2; EC = 1.16.1.7; Ferric-chelate reductase 2	3.591
Cg1g023140	Ferric-chelate reductase [EC:1.16.1.7] | (RefSeq) ferric reduction oxidase 7, chloroplastic-like (A)	NAC domain-containing protein 104 {ECO:0000305}	3.271
Cg2g021360	Ferric-chelate reductase [EC:1.16.1.7] | (RefSeq) ferric reduction oxidase 7, chloroplastic-like (A)	NAC domain-containing protein 72	2.012
Cg5g010410	Ferric-chelate reductase [EC:1.16.1.7] | (RefSeq) ferric reduction oxidase 7, chloroplastic-like (A)	NAC domain-containing protein 100 {ECO:0000303|PubMed:15029955}	1.441
Cg6g025130	Ferric-chelate reductase [EC:1.16.1.7] | (RefSeq) ferric reduction oxidase 7, chloroplastic-like (A)	NAC domain-containing protein 100 {ECO:0000303|PubMed:15029955}	1.777
Cg8g013730	Ferric-chelate reductase [EC:1.16.1.7] | (RefSeq) ferric reduction oxidase 7, chloroplastic-like (A)	NAC domain-containing protein 90	−1.805

FC: fold change.

## Data Availability

The raw transcriptome data have been deposited in NCBI SRA database (accession number: PRJNA702620). Data are archived in L.-S. Chen’s lab and available upon request.

## Supplementary Materials

The following are available online at https://www.mdpi.com/article/10.3390/ijms222112023/s1.

## References

[1] Adrees M., Ali S., Rizwan M., Ibrahim M., Abbas F., Farid M., Ziaurrehman M., Irshad M.K., Bharwana S.A. (2015). The effect of excess copper on growth and physiology of important food crops: A review. Environ. Sci. Pollut. Res..

[2] Li Q., Chen H.-H., Qi Y.-P., Ye X., Yang L.-T., Huang Z.-R., Chen L.-S. (2019). Excess copper effects on growth, uptake of water and nutrients, carbohydrates, and PSII photochemistry revealed by OJIP transients in *Citrus* seedlings. Environ. Sci. Pollut. Res..

[3] Cheng C., Zhang S.-Q., Lin W.-J., Chen H.-H., Lin F., Zhu D.-H., Chen L.-S., Li Y., Guo J.-X. (2018). Soil copper (Cu) nutrient status and its influencing factors in pomelo orchards in Pinghe county, Fujian Province. J. Fruit Sci..

[4] Li Y., Han M.-Q., Lin F., Ten Y., Lin J., Zhu D.-H., Guo P., Weng Y.-B., Chen L.-S. (2015). Soil chemical properties, ‘Guanximiyou’ pummelo leaf mineral nutrient status and fruit quality in the southern region of Fujian province, China. J. Soil Sci. Plant Nutr..

[5] Huang Y.Y., Liu B., Chen G.F., Wang Y. (2007). Content of copper in soil, *Citrus* leaves, and branch roots of *Citrus* orchards in Guangxi. Southwest China J. Agric. Sci..

[6] Alva A.K., Huang B., Prakash O., Paramasivam S. (1999). Effects of copper rates and soil pH on growth and nutrient uptake by *Citrus* seedlings. J. Plant Nutr..

[7] Kumar V., Pandita S., Sidhu G.P.S., Sharma A., Khanna K., Kaur P., Bali A.S., Setia R. (2021). Copper bioavailability, uptake, toxicity and tolerance in plants: A comprehensive review. Chemosphere.

[8] Wan H., Du J., He J., Lyu D., Li H. (2019). Copper accumulation, subcellular partitioning and physiological and molecular responses in relation to different copper tolerance in apple rootstocks. Tree Physiol..

[9] Shabbir Z., Sardar A., Shabbir A., Abbas G., Shamshad S., Khalid S., Natasha, Murtaza G., Dumat C., Shahid M. (2020). Copper uptake, essentiality, toxicity, detoxification and risk assessment in soil-plant environment. Chemosphere.

[10] Cai L.-Y., Zhang J., Ren Q.-Q., Lai Y.-H., Peng M.-Y., Deng C.-L., Ye X., Yang L.-T., Yang Z.-R., Chen L.-S. (2021). Increased pH-mediated alleviation of copper-toxicity and growth response function in *Citrus sinensis* seedlings. Sci. Hortic..

[11] Zhang H., Zhang F., Xia Y., Wang G., Shen Z. (2018). Excess copper induces production of hydrogen peroxide in the leaf of *Elsholtzia haichowensis* through apoplastic and symplastic CuZn-superoxide dismutase. J. Hazard. Mater..

[12] Hippler F.W.R., Boaretto R.M., Dovis V.L., Quaggio J.A., Azevedo R.A., Mattos D. (2018). Oxidative stress induced by Cu nutritional disorders in *Citrus* depends on nitrogen and calcium availability. Sci. Rep..

[13] Kováčik J., Klejdus B., Hedbavny J., Štork F., Bačkor M. (2009). Comparison of cadmium and copper effect on phenolic metabolism, mineral nutrients and stress-related parameters in *Matricaria chamomilla* plants. Plant Soil.

[14] Lequeux H., Hermans C., Lutts S., Verbruggen N. (2010). Response to copper excess in *Arabidopsis thaliana*: Impact on the root system architecture, hormone distribution, lignin accumulation and mineral profile. Plant Physiol. Biochem..

[15] Zhang L.L., He X.J., Chen M., An R.D., An X.L., Li J. (2014). Responses of nitrogen metabolism to copper stress in *Luffa cylindrica* roots. J. Soil Sci. Plant Nutr..

[16] Xia Y., Yin S., Zhang K., Shi X., Shen Z. (2018). Oswak11, a rice wall-associated kinase, regulates Cu detoxification by alteration the immobilization of cu in cell walls. Environ. Exp. Bot..

[17] Shi K., Liu X., Zhu Y., Bai Y., Shan D., Zheng X., Wang L., Zhang H., Wang C., Yan T. (2020). MdWRKY11 improves copper tolerance by directly promoting the expression of the copper transporter gene *MdHMA5*. Hortic. Res..

[18] Wan H., Yang F., Zhuang X., Cao Y., He J., Li H., Qin S., Lyu D. (2021). *Malus* rootstocks affect copper accumulation and tolerance in trees by regulating copper mobility, physiological responses, and gene expression patterns. Environ. Pollut..

[19] Wang Q.Y., Liu J.S., Hu B. (2016). Integration of copper subcellular distribution and chemical forms to understand copper toxicity in apple trees. Environ. Exp. Bot..

[20] Huang W.-L., Wu F.-L., Huang H.-Y., Huang W.-T., Deng C.-L., Yang L.-T., Huang Z.-R., Chen L.-S. (2020). Excess copper-induced alterations of protein profiles and related physiological parameters in *Citrus* leaves. Plants.

[21] Printz B., Lutts S., Hausman J.F., Sergeant K. (2016). Copper trafficking in plants and its implication on cell wall dynamics. Front. Plant Sci..

[22] Bouazizi H., Jouili H., Geitmann A., El Ferjani E. (2011). Cell wall accumulation of Cu ions and modulation of lignifying enzymes in primary leaves of bean seedlings exposed to excess copper. Biol. Trace Elem. Res..

[23] Vinit-Dunand F., Epron D., Alaoui-Sossé B., Badot P.M. (2002). Effects of copper on growth and on photosynthesis of mature and expanding leaves in cucumber plants. Plant Sci..

[24] Jalmi S.K., Bhagat P.K., Verma D., Noryang S., Tayyeba S., Singh K., Sharma D., Sinha A.K. (2018). Traversing the links between heavy metal stress and plant signaling. Front. Plant Sci..

[25] Thao N.P., Khan M.I., Thu N.B., Hoang X.L., Asgher M., Khan N.A., Tran L.S. (2015). Role of ethylene and its cross talk with other signaling molecules in plant responses to heavy metal stress. Plant Physiol..

[26] Zehra A., Choudhary S., Wani K.I., Naeem M., Khan M.M.A., Aftab T. (2020). Exogenous abscisic acid mediates ROS homeostasis and maintains glandular trichome to enhance artemisinin biosynthesis in *Artemisia annua* under copper toxicity. Plant Physiol. Biochem..

[27] Fariduddin Q., Yusuf M., Hayat S., Ahmad A. (2009). Effect of 28-homobrassinolide on antioxidant capacity and photosynthesis in *Brassica juncea* plants exposed to different levels of copper. Environ. Exp. Bot..

[28] Hu Z., Fu Q., Zheng J., Zhang A., Wang H. (2020). Transcriptomic and metabolomic analyses reveal that melatonin promotes melon root development under copper stress by inhibiting jasmonic acid biosynthesis. Horti. Res..

[29] Landa P., Dytrych P., Prerostova S., Petrova S., Vankova R., Vanek T. (2017). Transcriptomic response of *Arabidopsis thaliana* exposed to CuO nanoparticles, bulk material, and ionic copper. Environ. Sci. Technol..

[30] Lin C.Y., Trinh N.N., Fu S.F., Hsiung Y.C., Chia L.C., Lin C.W., Huang H.J. (2013). Comparison of early transcriptome responses to copper and cadmium in rice roots. Plant Mol. Biol..

[31] Zhang Z., Ke M., Qu Q., Peijnenburg W.J.G.M., Lu T., Zhang Q., Ye Y., Xu P., Du B., Sun L. (2018). Impact of copper nanoparticles and ionic copper exposure on wheat (*Triticum aestivum* L.) root morphology and antioxidant response. Environ. Pollut..

[32] Cao Y.Y., Qi C.D., Li S., Wang Z., Wang X., Wang J., Ren S., Li X., Zhang N., Guo Y.D. (2019). Melatonin alleviates copper toxicity via improving copper sequestration and ROS scavenging in cucumber. Plant Cell Physiol..

[33] Fu X.Z., Zhang X.Y., Qiu J.Y., Zhou X., Yuan M., He Y.Z., Chun C.P., Cao L., Ling L.L., Peng L.Z. (2019). Whole-transcriptome RNA sequencing reveals the global molecular responses and ceRNA regulatory network of mRNAs, lncRNAs, miRNAs and circRNAs in response to copper toxicity in Ziyang Xiangcheng (*Citrus junos* Sieb. Ex Tanaka). BMC Plant Biol..

[34] Leng X., Jia H., Sun X., Shangguan L., Wu Q., Wang B., Fang J. (2015). Comparative transcriptome analysis of grapevine in response to copper stress. Sci. Rep..

[35] Chen M., Fang X., Wang Z., Shangguan L., Liu T., Chen C., Liu Z., Ge M., Zhang C., Zheng T. (2021). Multi-omics analyses on the response mechanisms of ‘Shine Muscat’ grapevine to low degree of excess copper stress (Low-ECS). Environ. Pollut..

[36] Sudo E., Itouga M., Yoshida-Hatanaka K., Ono Y., Sakakibara H. (2008). Gene expression and sensitivity in response to copper stress in rice leaves. J. Exp. Bot..

[37] Guo P., Qi Y.-P., Huang W.-L., Yang L.-T., Huang Z.-R., Lai N.-W., Chen L.-S. (2018). Aluminum-responsive genes revealed by RNA-Seq and related physiological responses in leaves of two *Citrus* species with contrasting aluminum-tolerance. Ecotoxicol. Environ. Saf..

[38] Guo P., Qi Y.-P., Yang L.-T., Lai N.-W., Ye X., Yang Y., Chen L.-S. (2017). Root adaptive responses to aluminum-treatment revealed by RNA-Seq in two *Citrus* species with different aluminum-tolerance. Front. Plant Sci..

[39] Guo F., Yu H., Xu Q., Deng X. (2015). Transcriptomic analysis of differentially expressed genes in an orange-pericarp mutant and wild type in pummelo (*Citrus grandis*). BMC Plant Biol..

[40] Burkhead J.L., Reynolds K.A.G., Abdel-Ghany S.E., Cohu C.M., Pilon M. (2009). Copper homeostasis. New Phytol..

[41] Sancenón V., Puig S., Mira H., Thiele D.J., Peñarrubia L. (2003). Identification of a copper transporter family in *Arabidopsis thaliana*. Plant Mol. Biol..

[42] Klaumann S., Nickolaus S.D., Fürst S.H., Starck S., Schneider S., Neuhaus E.H., Trentmann O. (2011). The tonoplast copper transporter COPT5 acts as an exporter and is required for interorgan allocation of copper in *Arabidopsis thaliana*. New Phytol..

[43] Robinson N.J., Procter C.M., Connolly E.L., Guerinot M.L. (1999). A ferric-chelate reductase for iron uptake from soils. Nature.

[44] Welch R.M., Norvell W.A., Schaefer S.C., Shaff J.E., Kochian L.V. (1993). Induction of iron(III) and copper(II) reduction in pea roots by Fe and Cu status: Does the root-cell plasmalemma Fe(III)-chelate reductase perform a general role in regulation of cation uptake. Planta.

[45] Jeong J., Cohu C., Kerkeb L., Pilon M., Connolly E.L., Guerinot M.L. (2008). Chloroplast Fe(III) chelate reductase activity is essential for seedling viability under iron limiting conditions. Proc. Natl. Acad. Sci. USA.

[46] Zheng L., Yamaji N., Yokosho K., Ma J.F. (2012). YSL16 is a phloem-localized transporter of the copper-nicotianamine complex that is responsible for copper distribution in rice. Plant Cell.

[47] Blaby-Haas C.E., Padilla-Benavides T., Stübe R., Argüello J.M., Merchant S.S. (2014). Evolution of a plant-specific copper chaperone family for chloroplast copper homeostasis. Proc. Natl. Acad. Sci. USA.

[48] Del Pozo T., Cambiazo V., González M. (2010). Gene expression profiling analysis of copper homeostasis in *Arabidopsis thaliana*. Biochem. Bioph. Res. Commun..

[49] Schiavon M., Zhang L., Abdel-Ghany S.E., Pilon M., Malagoli M., Pilon-Smits E.A.H. (2007). Variation in copper tolerance in *Arabidopsis thaliana* accessions Columbia, Landsberg erecta and Wassilewskija. Physiol. Plant..

[50] Huang H.-Y., Ren Q.-Q., Lai Y.-H., Peng M.-Y., Zhang J., Yang L.-T., Huang Z.-R., Chen L.-S. (2021). Metabolomics combined with physiology and transcriptomics reveals how *Citrus grandis* leaves cope with copper-toxicity. Ecotoxicol. Environ. Saf..

[51] Hou X., Tong H., Selby J., Dewitt J., Peng X., He Z.H. (2005). Involvement of a cell wall-associated kinase, WAKL4, in *Arabidopsis* mineral responses. Plant Physiol..

[52] Hayashi T. (1989). Xyloglucans in the primary cell wall. Annu. Rev. Plant Physiol. Plant Mol. Biol..

[53] Zhu X.F., Shi Y.Z., Lei G.J., Fry S.C., Zhang B.C., Zhou Y.H., Braam J., Jiang T., Xu X.Y., Mao C.Z. (2012). XTH31, encoding an in vitro XEH/XET-active enzyme, controls Al sensitivity by modulating in vivo XET action, cell wall xyloglucan content and Al binding capacity in *Arabidopsis*. Plant Cell.

[54] Zhu X.F., Wan J.X., Sun Y., Shi Y.Z., Braam J., Li G.X., Zheng S.J. (2014). Xyloglucan endotransglucosylase-hydrolase17 interacts with xyloglucan endotransglucosylase-hydrolase31 to confer xyloglucan endotransglucosylase action and affect aluminum sensitivity in *Arabidopsis*. Plant Physiol..

[55] Jiang H.-X., Yang L.-T., Qi Y.-P., Lu Y.B., Huang Z.-R., Chen L.-S. (2015). Root iTRAQ protein profile analysis of two *Citrus* species differing in aluminum-tolerance in response to long-term aluminum-toxicity. BMC Genomics.

[56] Wang Z.Q., Xu X.Y., Gong Q.Q., Xie C., Fan W., Yang J.L., Lin Q.S., Zheng S.J. (2014). Root proteome of rice studied by iTRAQ provides integrated insight into aluminum stress tolerance mechanisms in plants. J. Proteomics.

[57] Asada K. (2000). 2000. The water-water cycle as alternative photon and electron sinks. Phil. Trans. R. Soc. Lond. B.

[58] Cohu C.M., Pilon M. (2007). Regulation of superoxide dismutase expression by copper availability. Physiol. Plant..

[59] Abdel-Ghany S.E., Burkhead J.L., Gogolin K.A., Andrés-Colás N., Bodecker J.R., Puig S., Peñarrubia L., Pilon M. (2005). AtCCS is a functional homolog of the yeast copper chaperone Ccs1/Lys7. FEBS Let..

[60] Huang C.-H., Kuo W.-Y., Weiss C., Jinn T.L. (2012). Copper chaperone-dependent and -independent activation of three copper-zinc superoxide dismutase homologs localized in different cellular compartments in *Arabidopsis*. Plant Physiol..

[61] Davletova S., Rizhsky L., Liang H., Zhong S., Oliver D.J., Coutu J., Shulaev V., Schlauch K., Mittleret R. (2005). Cytosolic ascorbate peroxidase 1 is a central component of the reactive oxygen gene network of *Arabidopsis*. Plant Cell.

[62] Lim J.D., Hahn S.J., Yu C.Y., Chung I.M. (2015). Expression of the glutathione S-transferase gene (NT107) in transgenic *Dianthus superbus*. Plant Cell Tissue Organ Cult..

[63] Zhang L., Du L., Poovaiah B.W. (2014). Calcium signaling and biotic defense responses in plants. Plant Signal. Behav..

[64] Krebs J., Agellon L.B., Michalak M. (2015). Ca^2+^ homeostasis and endoplasmic reticulum (ER) stress: An integrated view of calcium signaling. Biochem. Bioph. Res. Co..

[65] Teardo E., Carraretto L., De Bortoli S., Costa A., Behera S., Wagner R., Lo Schiavo F., Formentin E., Szabo I. (2015). Alternative splicing-mediated targeting of the *Arabidopsis* glutamate receptor 3.5 to mitochondria affects organelle morphology. Plant Physiol..

[66] Teardo E., Formentin E., Segalla A., Giacometti G.M., Marin O., Zanetti M., Lo Schiavo F., Zoratti M., Szabò I. (2011). Dual localization of plant glutamate receptor AtGLR3.4 to plastids and plasmamembrane. Biochim. Biophys. Acta-Bioenergetics.

[67] Yan J., Guan L., Sun Y., Zhu Y., Liu L., Lu R., Jiang M., Tan M., Zhang A. (2015). Calcium and ZmCCaMK are involved in brassinosteroid-induced antioxidant defense in maize leaves. Plant Cell Physiol..

[68] Ma F., Lu R., Liu H., Shi B., Zhang J., Tan M., Zhang A., Jiang M. (2012). Nitric oxide-activated calcium/calmodulin-dependent protein kinase regulates the abscisic acid-induced antioxidant defence in maize. J. Exp. Bot..

[69] Pearce G., Moura D.S., Stratmann J., Ryan C.A. (2001). RALF, a 5-kDa ubiquitous polypeptide in plants, arrests root growth and development. Proc. Natl. Acad. Sci. USA.

[70] Zhao C., Zayed O., Yu Z., Jiang W., Zhu P., Hsu C.C., Zhang L., Tao W.A., Lozano-Durán R., Zhu J.K. (2018). Leucine-rich repeat extensin proteins regulate plant salt tolerance in *Arabidopsis*. Proc. Natl. Acad. Sci. USA.

[71] Yeh C.M., Chine P.S., Huang H.J. (2007). Distinct signalling pathways for induction of MAP kinase activities by cadmium and copper in rice roots. J. Exp. Bot..

[72] Burnett E.C., Desikan R., Moser R.C., Neill S.J. (2000). ABA activation of an MBP kinase in *Pisum sativum* epidermal peels correlates with stomatal responses to ABA. J. Exp. Bot..

[73] Agrawal G.K., Agrawal S.K., Shibato J., Iwahashi H., Rakwal R. (2003). Novel rice MAP kinases OsMSRMK3 and OsWJUMK1 involved in encountering diverse environmental stresses and developmental regulation. Biochem. Biophys. Res. Commun..

[74] Schweighofer A., Hirt H., Meskiene I. (2004). Plant PP2C phosphatases: Emerging functions in stress signaling. Trends Plant Sci..

[75] Singh A., Johan S.K., Bagri J., Pandey G.K. (2015). ABA inducible rice protein phosphatase 2C confers ABA insensitivity and abiotic stress tolerance in *Arabidopsis*. PLoS ONE.

[76] Liu X., Zhu Y., Zhai H., Cai H., Ji W., Luo X., Li J., Bai X. (2012). AtPP2CG1, a protein phosphatase 2C, positively regulates salt tolerance of *Arabidopsis* in abscisic acid-dependent manner. Biochem. Biophys. Res. Commun..

[77] Ouzounidou G., Ilias I. (2005). Hormone-induced protection of sunflower photosynthetic apparatus against copper toxicity. Biol. Plant..

[78] Xiong L., Lee H., Ishitani M., Zhu J.K. (2002). Regulation of osmotic stress-responsive gene expression by the *LOS6/ABA1* locus in *Arabidopsis*. J. Biol. Chem..

[79] Wang Y., Wang Y., Kai W., Zhao B., Chen P., Sun L., Ji K., Li Q., Dai S., Sun Y. (2014). Transcriptional regulation of abscisic acid signal core components during cucumber seed germination and under Cu^2+^, Zn^2+^, NaCl and simulated acid rain stresses. Plant Physiol. Biochem..

[80] Zengin F.K., Kirbag S. (2007). Effects of copper on chlorophyll, proline, protein and abscisic acid level of sunflower (*Helianthus annuus* L.) seedlings. J. Environ. Biol..

[81] Nguyen T.Q., Sesin V., Kisiala A., Emery R.J.N. (2021). Phytohormonal roles in plant responses to heavy metal stress: Implications for using macrophytes in phytoremediation of aquatic ecosystems. Environ. Toxicol. Chem..

[82] Kuroha T., Tokunaga H., Kojima M., Ueda N., Ishida T., Nagawa S., Fukuda H., Sugimoto K., Sakakibara H. (2009). Functional analyses of LONELY GUY cytokinin-activating enzymes reveal the importance of the direct activation pathway in *Arabidopsis*. Plant Cell.

[83] Thomas J.C., Perron M., LaRosa P.C., Smigocki A.C. (2005). Cytokinin and the regulation of a tobacco metallothionein-like gene during copper stress. Physiol. Plant..

[84] Yang T.-Y., Cai L.-Y., Qi Y.-P., Yang L.-T., Lai N.-W., Chen L.-S. (2019). Increasing nutrient solution pH alleviated aluminum-induced inhibition of growth and impairment of photosynthetic electron transport chain in *Citrus sinensis* seedlings. BioMed. Res. Int..

[85] Guo P., Qi Y.-P., Cai Y.-T., Yang T.-Y., Yang L.-T., Huang Z.-R., Chen L.-S. (2018). Aluminum effects on photosynthesis, reactive oxygen species and methylglyoxal detoxification in two *Citrus* species differing in aluminum tolerance. Tree Physiol..

[86] Lichtenthaler H.K. (1987). Chlorophylls and carotenoids: Pigments of photosynthetic biomembranes. Methods Enzymol..

[87] Long A., Zhang J., Yang L.-T., Ye X., Lai N.-W., Tan L.-L., Lin D., Chen L.-S. (2017). Effects of low pH on photosynthesis, related physiological parameters and nutrient profile of *Citrus*. Front. Plant Sci..

[88] Garg N., Kaur H. (2013). Response of antioxidant enzymes, phytochelatins and glutathione production towards Cd and Zn stresses in *Cajanus cajan* (L.) Millsp. genotypes colonized by arbuscular mycorrhizal fungi. J. Agron. Crop Sci..

[89] Malik J.A., Goel S., Kaur N., Sharma S., Singh I., Nayyar H. (2012). Selenium antagonises the toxic effects of arsenic on mungbean (*Phaseolus aureus* Roxb.) plants by restricting its uptake and enhancing the antioxidative and detoxification mechanisms. Environ. Exp. Bot..

[90] Chen S., Zhou Y., Chen Y., Gu J. (2018). fastp: An ultra-fast all-in-one FASTQ preprocessor. Bioinformatics.

[91] Pertea M., Pertea G.M., Antonescu C.M., Chang T.C., Mendell J.T., Salzberg S.L. (2015). StringTie enables improved reconstruction of a transcriptome from RNA-seq reads. Nature Biotechnol..

[92] Liao Y., Smyth G.K., Shi W. (2014). FeatureCounts: An efficient general purpose program for assigning sequence reads to genomic features. Bioinformatics.

[93] Chen L., Wu Q., He W., He T., Wu Q., Miao Y. (2019). Combined *de novo* transcriptome and metabolome analysis of common bean response to *Fusarium oxysporum* f. sp. phaseoli infection. Int. J. Mol. Sci..

[94] Mostafa H.H.A., Wang H., Song J., Li X. (2020). Effects of genotypes and explants on garlic callus production and endogenous hormones. Sci. Rep..

