# Supplementary material for: Adaptive Responses of Citrus grandis Leaves to Copper Toxicity Revealed by RNA-Seq and Physiology
Source: Int J Mol Sci. 2021 Nov 6;22(21):12023. doi: 10.3390/ijms222112023 (PMC8585100; doi:10.3390/ijms222112023)
Supplement: Supplementary file 1 [file ijms-22-12023-s001.zip › 2012WUIJMSSupplementaryData/2021WuIJMSFigure S1.pdf]

Figure S1

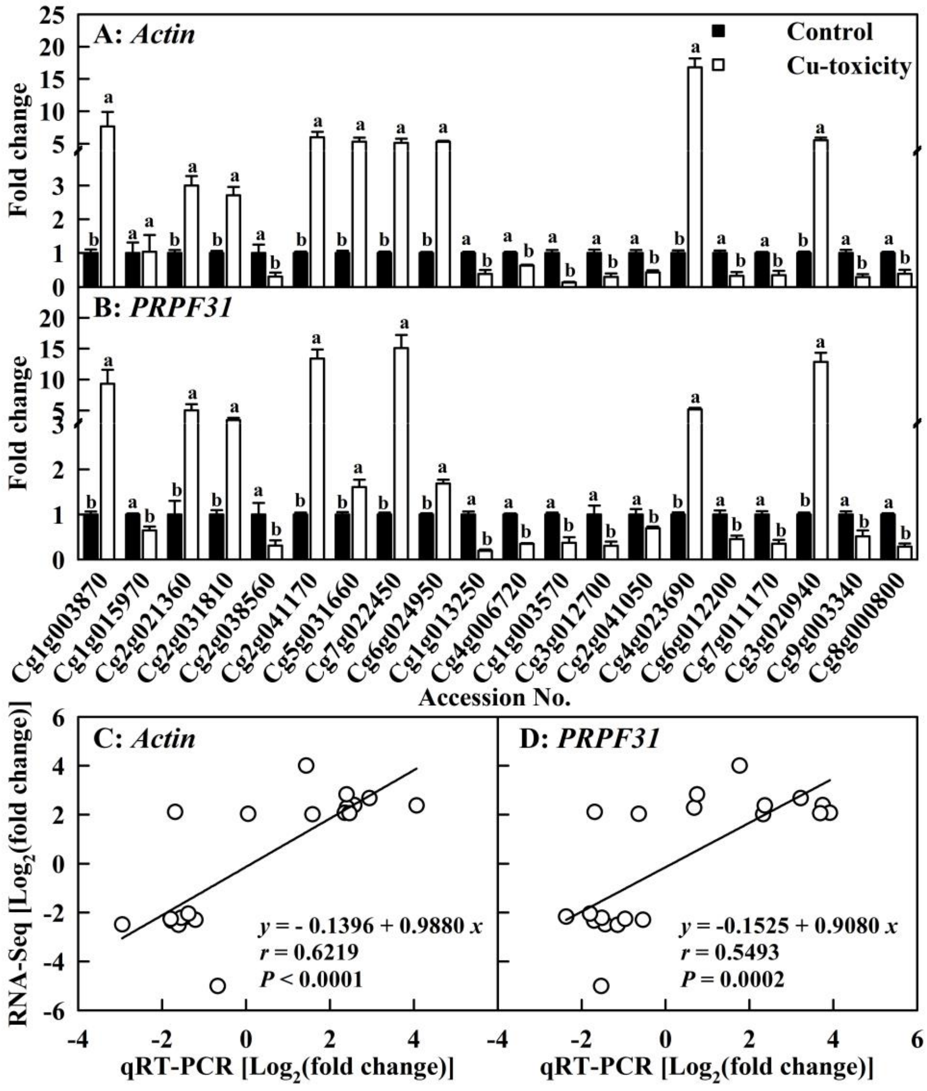

Figure S1. qRT-PCR validation of 20 DEGs using *actin* (**A**) and *PRPF31* (**B**) as internal standards, and the correlation analysis of RNA-Seq data and qRT-PCR results using *actin* (**C**) and PRPF31 (**D**) as internal standards. Bars represent means ± SE of 2 technique and 3 biological replicates. For the same genes, different letters above the bars indicate a significant difference at $P < 0.05$. For (**C**) and (**D**), RNA-Seq data came from Table S5.
