# Supplementary material for: Adaptive Responses of Citrus grandis Leaves to Copper Toxicity Revealed by RNA-Seq and Physiology
Source: Int J Mol Sci. 2021 Nov 6;22(21):12023. doi: 10.3390/ijms222112023 (PMC8585100; doi:10.3390/ijms222112023)
Supplement: Supplementary file 1 [file ijms-22-12023-s001.zip › 2012WUIJMSSupplementaryData/2021WuIJMSTables S1-S2.pdf]

Tables S1-S2

Table S1. Summary of the RNA-Seq data collected from leaves of *C. grandis* seedlings treated with 0.5 (control) and 400 (Cu-toxicity) μM Cu. Q20 and Q30 mean sequencing error rates lower than 1% and 1‰, respectively.

Samples treatment	Raw reads	Clean reads (%)	Clean bases (G)	Error rate (%)	Q20 (%)	Q30 (%)	Adapter (%)	N (%)	Low quality (%)	GC content (%)
Control 1	47326492	46291438 (97.8)	6.94	0.02	98.09	94.24	2.06	0.003	0.12	43.44
Control 2	54676210	53395986 (97.7)	8.01	0.02	98.12	94.42	2.21	0.003	0.13	43.32
Control 3	49156910	47471830 (96.6)	7.12	0.02	98.03	94.07	3.32	0.003	0.10	43.42
Cu-toxicity 1	51570710	50549974 (98.0)	7.58	0.02	97.5	92.73	1.90	0.000	0.08	43.63
Cu-toxicity 2	54513246	53349850 (97.9)	8.00	0.02	97.11	91.9	2.01	0.005	0.12	43.55
Cu-toxicity 3	55041176	53936214 (98.0	8.09	0.02	97.22	92.12	1.89	0.005	0.11	43.78

Table S2. Summary of clean reads and genes mapped to the reference genome from leaves of *C. grandis* seedlings treated with 0.5 and 400 μM Cu.

Samples	Total Reads	Reads mapped	Unique mapped	Multi mapped	Read1 mapped	Read2 mapped	'+' mapped	'-' mapped
Control 1	46291438	43438909 (93.84%)	42075051 (90.89%)	1998815 (2.95%)	20941450 (45.24%)	21133601 (45.65%)	21037984(45.45%)	21037067 (45.44%)
Control 2	53395986	49960804 (93.57%)	48383775 (90.61%)	2359390 (2.95%)	23999749 (44.95%)	24384026 (45.67%)	24194985(45.31%)	24188790 (45.30%)
Control 3	47471830	44549092 (93.84%)	43209659 (91.02%)	1910286 (2.82%)	21508433 (45.31%)	21701226 (45.71%)	21599098(45.50%)	21610561 (45.52%)
Cu-toxicity 1	50549974	48205117 (95.36%)	46793588 (92.57%)	2059646 (2.79%)	23443675 (46.38%)	23349913 (46.19%)	23381225(46.25%)	23412363 (46.32%)
Cu-toxicity 2	53349850	50499175 (94.66%)	48983206 (91.82%)	2299373 (2.84%)	24605800 (46.12%)	24377406 (45.69%)	24466437(45.86%)	24516769 (45.95%)
Cu-toxicity 3	53936214	51150060 (94.83%)	49625548 (92.01%)	2257448 (2.83%)	24911486 (46.19%)	24714062 (45.82%)	24784662(45.95%)	24840886(46.06%)
