# Supplementary material for: Adaptive Responses of Citrus grandis Leaves to Copper Toxicity Revealed by RNA-Seq and Physiology
Source: Int J Mol Sci. 2021 Nov 6;22(21):12023. doi: 10.3390/ijms222112023 (PMC8585100; doi:10.3390/ijms222112023)
Supplement: Supplementary file 1 [file ijms-22-12023-s001.zip › 2012WUIJMSSupplementaryData/2021WuIJMSTables S9-S18.pdf]

Tables S9-18

Table S9. DEGs involved in cell well metabolism in leaves of *C. grandis* seedlings exposed to Cu-toxicity

Accession No.	KEGG	Swiss-Prot	$Log_2(FC)$
Plant-type cell wall (GO:0009505)			
Cg1g015520	LRR receptor-like serine/threonine-protein kinase FLS2 [EC:2.7.11.1]	LRR receptor-like serine/threonine-protein kinase GSO2	-1.602
Cg4g008420	LRR receptor-like serine/threonine-protein kinase FLS2 [EC:2.7.11.1]	Leucine-rich repeat extensin-like protein 4	-1.176
Cg2g024270	Interleukin-1 receptor-associated kinase 4 [EC:2.7.11.1]	Wall-associated receptor kinase-like 2	-1.330
Cg1g024330	Beta-galactosidase [EC:3.2.1.23]	Beta-galactosidase 10	-1.209
Cg3g014760	Nucleoporin SEH1	Protein EXORDIUM-like 2	-2.037
CgUng004910	Nucleoporin SEH1	Protein EXORDIUM-like 2	-2.760
Cg4g019830	Heparanase [EC:3.2.1.166]	Heparanase-like protein 3	-1.388
Cg5g008280	Saccharopepsin [EC:3.4.23.25]	Probable aspartyl protease At4g16563 {ECO:0000305}	-1.082
Cg7g011170	Alpha-mannosidase [EC:3.2.1.24]	Probable alpha-mannosidase At5g13980 {ECO:0000305}	-2.225
Cg7g014460	Alpha-D-xyloside xylohydrolase [EC:3.2.1.177]	Alpha-xylosidase 1	-1.159
Cg9g001580	Xylan 1,4-beta-xylosidase [EC:3.2.1.37]	Beta-D-xylosidase 1	-1.581
Cg5g038180	Xylan 1,4-beta-xylosidase [EC:3.2.1.37]	Probable beta-D-xylosidase 2	-1.672
Cg9g026970	O-palmitoleoyl-L-serine hydrolase [EC:3.1.1.98]	Pectin acetylesterase 9 {ECO:0000303|PubMed:25115560}	-2.264
Cg9g002460	Sucrose synthase [EC:2.4.1.13]	Sucrose synthase 2	3.483
Cell wall organization (GO:0071555)			
Cg1g015500	LRR receptor-like serine/threonine-protein kinase FLS2 [EC:2.7.11.1]	LRR receptor-like serine/threonine-protein kinase FLS2	-1.275
Cg1g015520	LRR receptor-like serine/threonine-protein kinase FLS2 [EC:2.7.11.1]	LRR receptor-like serine/threonine-protein kinase GSO2	-1.602
Cg4g008420	LRR receptor-like serine/threonine-protein kinase FLS2 [EC:2.7.11.1]	Leucine-rich repeat extensin-like protein 4	-1.176
Cg9g006130	LRR receptor-like serine/threonine-protein kinase FLS2 [EC:2.7.11.1]	Leucine-rich repeat extensin-like protein 6	-2.603
Cg2g040340	Protein transport protein SEC61 subunit alpha	COBRA-like protein 10	-2.721
CgUng006010	Leucine-rich PPR motif-containing protein, mitochondrial	COBRA-like protein 1	9.115
Cg3g023440	Integrin-linked kinase-associated serine/threonine phosphatase 2C [EC:3.1.3.16]	Protein WALLS ARE THIN 1	2.919
Cg4g000800	Xyloglucan glycosyltransferase 4 [EC:2.4.1.-]	Probable xyloglucan glycosyltransferase 5	3.743
Cg1g007100	Xyloglucan:xyloglucosyl transferase [EC:2.4.1.207]	Probable xyloglucan endotransglucosylase/hydrolase protein 6	-1.880
Cg2g017560	Xyloglucan:xyloglucosyl transferase [EC:2.4.1.207]	Xyloglucan endotransglucosylase/hydrolase protein 9	-2.685
Cg2g020180	Omega-hydroxypalmitate O-feruloyl transferase [EC:2.3.1.188]	Omega-hydroxypalmitate O-feruloyl transferase	-3.290

Cg2g031930	F-box and leucine-rich repeat protein 2/20	Putative pectinesterase/pectinesterase inhibitor 22	-1.492
Cg1g013250	Pectinesterase [EC:3.1.1.11]	Pectinesterase 3	-2.176
Cg2g033370	Pectinesterase [EC:3.1.1.11]	Probable pectinesterase/pectinesterase inhibitor 6	-1.299
Cg4g018220	Pectinesterase [EC:3.1.1.11]	Probable pectinesterase/pectinesterase inhibitor 54	-2.108
Cg5g018160	Pectinesterase [EC:3.1.1.11]	Probable pectinesterase/pectinesterase inhibitor 61	-1.288
Cg9g026970	O-palmitoleoyl-L-serine hydrolase [EC:3.1.1.98]	Pectin acetylesterase 9 {ECO:0000303|PubMed:25115560}	-2.264
Cg2g040360	Pectinesterase [EC:3.1.1.11]	Pectinesterase 2.2	1.647
Cg2g044240	DNA-directed RNA polymerase II subunit RPB1 [EC:2.7.7.6]	Glucan endo-1,3-beta-glucosidase 12	2.251
Cg8g014060	Glucan endo-1,3-beta-glucosidase 1/2/3 [EC:3.2.1.39]	Glucan endo-1,3-beta-glucosidase 14	1.055
Cg3g023860	Endoglucanase [EC:3.2.1.4]	Endoglucanase 10	1.121
Cg7g014460	Alpha-D-xyloside xylohydrolase [EC:3.2.1.177]	Alpha-xylosidase 1	-1.159
Cg5g009980	UDP-glucose 4-epimerase [EC:5.1.3.2]	Bifunctional UDP-glucose 4-epimerase and UDP-xylose 4-epimerase 1	-1.862
Cg5g000440	Abhydrolase domain-containing protein 5 [EC:2.3.1.51]	Probable lysophospholipase BODYGUARD 3 {ECO:0000303|PubMed:16415209}	7.905
Cg6g008750	Abhydrolase domain-containing protein 5 [EC:2.3.1.51]	Probable lysophospholipase BODYGUARD 4 {ECO:0000303|PubMed:16415209}	2.244
Cg5g030310	Cytochrome P450 family 71 subfamily A	Cytochrome P450 83B1	3.608
Cg7g014840	Probable galacturonosyltransferase-like 1 [EC:2.4.1.-]	Probable galacturonosyltransferase-like 3	-1.814
Cg7g019950	N-hydroxythioamide S-beta-glucosyltransferase [EC:2.4.1.195]	UDP-glycosyltransferase 74B1	2.549
Cg7g021600	Golgi-specific brefeldin A-resistance guanine nucleotide exchange factor 1	ARF guanine-nucleotide exchange factor GNOM	1.069
Cg9g007480	Cellulose synthase A [EC:2.4.1.12]	Cellulose synthase-like protein G2	1.063
Cell wall modification (GO:0042545)			
Cg1g015500	LRR receptor-like serine/threonine-protein kinase FLS2 [EC:2.7.11.1]	LRR receptor-like serine/threonine-protein kinase FLS2	-1.275
Cg2g031930	F-box and leucine-rich repeat protein 2/20	Putative pectinesterase/pectinesterase inhibitor 22	-1.492
Cg1g013250	Pectinesterase [EC:3.1.1.11]	Pectinesterase 3	-2.176
Cg2g033370	Pectinesterase [EC:3.1.1.11]	Probable pectinesterase/pectinesterase inhibitor 6	-1.299
Cg4g018220	Pectinesterase [EC:3.1.1.11]	Probable pectinesterase/pectinesterase inhibitor 54	-2.108
Cg5g018160	Pectinesterase [EC:3.1.1.11]	Probable pectinesterase/pectinesterase inhibitor 61	-1.288
Cg2g040360	Pectinesterase [EC:3.1.1.11]	Pectinesterase 2.2	1.647

Cg5g030310	Cytochrome P450 family 71 subfamily A	Cytochrome P450 83B1	3.608
Cg7g019950	N-hydroxythioamide S-beta-glucosyltransferase [EC:2.4.1.195]	UDP-glycosyltransferase 74B1	2.549
Cell wall macromolecule metabolic process (GO:0044036)			
Cg1g000850	Basic endochitinase B [EC:3.2.1.14]	Basic endochitinase	-3.753
Cg8g000790	Basic endochitinase B [EC:3.2.1.14]	Endochitinase	-4.533
Cg8g000800	Basic endochitinase B [EC:3.2.1.14]	Endochitinase	-4.480
Cg5g026680	Chitinase [EC:3.2.1.14]	Chitinase 4	4.598
Cg1g007100	Xyloglucan:xyloglucosyl transferase [EC:2.4.1.207]	Probable xyloglucan endotransglucosylase/hydrolase protein 6	-1.880
Cg2g017560	Xyloglucan:xyloglucosyl transferase [EC:2.4.1.207]	Xyloglucan endotransglucosylase/hydrolase protein 9	-2.685
Cg2g020180	Omega-hydroxypalmitate O-feruloyl transferase [EC:2.3.1.188]	Omega-hydroxypalmitate O-feruloyl transferase	-3.290
Cg7g014460	Alpha-D-xyloside xylohydrolase [EC:3.2.1.177]	Alpha-xylosidase 1	-1.159
Cg5g039550	Mannan endo-1,4-beta-mannosidase [EC:3.2.1.78]	Mannan endo-1,4-beta-mannosidase 4	-1.919
Cg6g019480	Mannose-6-phosphate isomerase [EC:5.3.1.8] ǀ	Mannose-6-phosphate isomerase 1	1.247
Cell wall pectin biosynthetic process (GO:0052325)			
Cg2g020180	Omega-hydroxypalmitate O-feruloyl transferase [EC:2.3.1.188]	Omega-hydroxypalmitate O-feruloyl transferase	-3.290
Callose deposition in cell wall (GO:0052543)			
Cg1g015500	LRR receptor-like serine/threonine-protein kinase FLS2 [EC:2.7.11.1]	LRR receptor-like serine/threonine-protein kinase FLS2	-1.275
Cg5g030310	Cytochrome P450 family 71 subfamily A	Cytochrome P450 83B1	3.608
Cg7g019950	N-hydroxythioamide S-beta-glucosyltransferase [EC:2.4.1.195]	UDP-glycosyltransferase 74B1	2.549
Cell wall thickening (GO:0052386)			
Cg1g015500	LRR receptor-like serine/threonine-protein kinase FLS2 [EC:2.7.11.1]	LRR receptor-like serine/threonine-protein kinase FLS2	-1.275
Cg5g030310	Cytochrome P450 family 71 subfamily A	Cytochrome P450 83B1	3.608
Cg7g019950	N-hydroxythioamide S-beta-glucosyltransferase [EC:2.4.1.195]	UDP-glycosyltransferase 74B1	2.549
Cell wall biogenesis (GO:0042546)			
Cg1g007100	Xyloglucan:xyloglucosyl transferase [EC:2.4.1.207]	Probable xyloglucan endotransglucosylase/hydrolase protein 6	-1.880
Cg2g017560	Xyloglucan:xyloglucosyl transferase [EC:2.4.1.207]	Xyloglucan endotransglucosylase/hydrolase protein 9	-2.685
Cg2g020180	Omega-hydroxypalmitate O-feruloyl transferase [EC:2.3.1.188]	Omega-hydroxypalmitate O-feruloyl transferase	-3.290
Cg2g040340	Protein transport protein SEC61 subunit alpha	COBRA-like protein 10	-2.721
Cg6g019480	Mannose-6-phosphate isomerase [EC:5.3.1.8] ǀ	Mannose-6-phosphate isomerase 1	1.247
Cg9g007480	Cellulose synthase A [EC:2.4.1.12]	Cellulose synthase-like protein G2	1.063
CgUng006010	Leucine-rich PPR motif-containing protein, mitochondrial	COBRA-like protein 1	9.115

Cg3g023440	Integrin-linked kinase-associated serine/threonine phosphatase 2C [EC:3.1.3.16]	Protein WALLS ARE THIN 1	2.919
Cg2g007490	Glucuronokinase [EC:2.7.1.43]	Glucuronokinase 1	1.774
	Plant-type cell wall organization or biogenesis (GO:0071669)		
Cg2g020180	Omega-hydroxypalmitate O-feruloyl transferase [EC:2.3.1.188]	Omega-hydroxypalmitate O-feruloyl transferase	-3.290
Cg2g040340	Protein transport protein SEC61 subunit alpha	COBRA-like protein 10	-2.721
CgUng006010	Leucine-rich PPR motif-containing protein, mitochondrial	COBRA-like protein 1	9.115
Cg3g013250	Branched-chain amino acid aminotransferase [EC:2.6.1.42]	Branched-chain-amino-acid aminotransferase 2, chloroplastic	1.973
Cg3g023440	Integrin-linked kinase-associated serine/threonine phosphatase 2C [EC:3.1.3.16]	Protein WALLS ARE THIN 1	2.919
Cg9g007480	Cellulose synthase A [EC:2.4.1.12]	Cellulose synthase-like protein G2	1.063
	Other		
Cg4g022640	Expansin	EG45-like domain containing protein 2	-4.198
Cg1g015970	Interleukin-1 receptor-associated kinase 4 [EC:2.7.11.1]	Wall-associated receptor kinase-like 10	2.024
Cg6g000930	Interleukin-1 receptor-associated kinase 4 [EC:2.7.11.1] | (RefSeq) putative wall-associated receptor kinase-like 16 (A)	Protein LYK5	6.121
Cg5g026300	Interleukin-1 receptor-associated kinase 4 [EC:2.7.11.1]	Wall-associated receptor kinase 5	-3.253

Table S10. DEGs related to stomata, chlorophyll and carotenoids metabolism, and photosynthesis in leaves of *C. grandis* seedlings exposed to Cu-toxicity

Accession No.	KEGG	Swiss-Prot	Log_2(FC)
Stomatal opening (GO:1990069)			
Cg8g019250	RING finger and CHY zinc finger domain-containing protein 1 [EC:2.3.2.27]	E3 ubiquitin-protein ligase RZFP34 {ECO:0000305}	-2.350
Stomatal closure (GO:0090332)			
Cg7g004470	Heat shock 70kDa protein 1/2/6/8	Probable mediator of RNA polymerase II transcription subunit 37e	4.597
Chlorophyll biosynthetic process (GO:0015995)			
Cg2g021210	Protochlorophyllide reductase [EC:1.3.1.33]	Protochlorophyllide reductase, chloroplastic	-1.721
Cg5g028200		Tetrapyrrole-binding protein, chloroplastic	-2.052
Cg6g017060	Magnesium-protoporphyrin IX monomethyl ester (oxidative) cyclase [EC:1.14.13.81]	Magnesium-protoporphyrin IX monomethyl ester [oxidative] cyclase, chloroplastic	-1.080
Cg9g006330	Mitochondrial pyruvate carrier 2	Early light-induced protein 1, chloroplastic {ECO:0000305}	1.715
Chlorophyll catabolic process (GO:0015996)			
Cg5g009510	Magnesium dechelatase [EC:4.99.1.10]	Protein STAY-GREEN 1, chloroplastic	1.601
Cg5g020710	Chlorophyllase [EC:3.1.1.14]		2.019
Carotenoid biosynthetic process (GO:0016117)			
Cg6g018390	Geranylgeranyl diphosphate synthase, type II [EC:2.5.1.1 2.5.1.10 2.5.1.29]	Geranylgeranyl pyrophosphate synthase, chloroplastic	-1.239
Photosynthesis (ko00195)			
Cg9g013190	F-type H^+-transporting ATPase subunit b	Putative ATP synthase protein YMF19	-1.145
Cg3g024680	Plastocyanin	Plastocyanin, chloroplastic	-1.448
Cg9g005290	Photosystem I subunit II	Photosystem I reaction center subunit II, chloroplastic	-1.501
Cg2g038220	Photosystem I subunit III	Photosystem I reaction center subunit III, chloroplastic	-1.014
Cg1g024920	Photosystem I subunit PsaN	Photosystem I reaction center subunit N, chloroplastic	-1.265
Cg7g005010	Photosystem II oxygen-evolving enhancer protein 2	PsbP-like protein 1, chloroplastic	-1.142
Cg7g012220	Photosystem II oxygen-evolving enhancer protein 2	Oxygen-evolving enhancer protein 2, chloroplastic	-1.749
Cg7g017970	Photosystem II PsbY protein	Photosystem II core complex proteins psbY, chloroplastic	-1.122
Cg2g040770	Photosystem II oxygen-evolving enhancer protein 3	Oxygen-evolving enhancer protein 3, chloroplastic	-2.463
Cg2g014580	Photosystem II Psb27 protein	Photosystem II repair protein PSB27-H1, chloroplastic	-1.149
Cg4g017260	Photosystem I subunit V	Photosystem I reaction center subunit V, chloroplastic	-1.799
Cg8g013250	Photosystem I subunit PsaO	Photosystem I subunit O	-2.645
Cg8g014070	Ferredoxin	Ferredoxin-3, chloroplastic	1.202
Photosynthesis- antenna proteins (ko00196)			
Cg7g006420	Light-harvesting complex I chlorophyll	Chlorophyll a-b binding protein 6A,	-1.626

	a/b binding protein 1	chloroplastic	
Cg2g044430	Light-harvesting complex II chlorophyll a/b binding protein 1	Chlorophyll a-b binding protein 3C, chloroplastic	-1.333
Cg2g044450	Light-harvesting complex II chlorophyll a/b binding protein 1	Chlorophyll a-b binding protein 3C, chloroplastic	-1.625
Cg2g020050	Light-harvesting complex II chlorophyll a/b binding protein 2	Chlorophyll a-b binding protein 151, chloroplastic	-1.880
Cg2g021790	Light-harvesting complex II chlorophyll a/b binding protein 3	Chlorophyll a-b binding protein 13, chloroplastic	-1.361
Cg6g006850	Light-harvesting complex II chlorophyll a/b binding protein 4	Chlorophyll a-b binding protein CP29.2, chloroplastic	-1.890
Cg5g023620	Light-harvesting complex II chlorophyll a/b binding protein 5	Chlorophyll a-b binding protein CP26, chloroplastic	-1.740
Cg2g025410	Light-harvesting complex II chlorophyll a/b binding protein 6	Chlorophyll a-b binding protein CP24 10B, chloroplastic	-1.234
Photosystem I (GO:0009522)			
Cg1g024920	Photosystem I subunit PsaN	Photosystem I reaction center subunit N, chloroplastic	-1.265
Cg2g020050	Light-harvesting complex II chlorophyll a/b binding protein 2	Chlorophyll a-b binding protein 151, chloroplastic	-1.880
Cg2g021790	Light-harvesting complex II chlorophyll a/b binding protein 3	Chlorophyll a-b binding protein 13, chloroplastic	-1.361
Cg2g038220	Photosystem I subunit III	Photosystem I reaction center subunit III, chloroplastic	-1.014
Cg2g044430	Light-harvesting complex II chlorophyll a/b binding protein 1	Chlorophyll a-b binding protein 3C, chloroplastic	-1.333
Cg2g044450	Light-harvesting complex II chlorophyll a/b binding protein 1	Chlorophyll a-b binding protein 3C, chloroplastic	-1.625
Cg4g017260	Photosystem I subunit V	Photosystem I reaction center subunit V, chloroplastic	-1.799
Cg5g023620	Light-harvesting complex II chlorophyll a/b binding protein 5	Chlorophyll a-b binding protein CP26, chloroplastic	-1.740
Cg6g006850	Light-harvesting complex II chlorophyll a/b binding protein 4	Chlorophyll a-b binding protein CP29.2, chloroplastic	-1.890
Cg7g006420	Light-harvesting complex I chlorophyll a/b binding protein 1	Chlorophyll a-b binding protein 6A, chloroplastic	-1.626
Cg8g013250	Photosystem I subunit PsaO	Photosystem I subunit O	-2.645
Cg9g005290	Photosystem I subunit II	Photosystem I reaction center subunit II, chloroplastic	-1.501
Cg9g006330	Mitochondrial pyruvate carrier 2	Early light-induced protein 1, chloroplastic {ECO:0000305}	1.715
Photosystem II (GO:0009523)			
Cg2g020050	Light-harvesting complex II chlorophyll a/b binding protein 2	Chlorophyll a-b binding protein 151, chloroplastic	-1.880
Cg2g021790	Light-harvesting complex II chlorophyll a/b binding protein 3	Chlorophyll a-b binding protein 13, chloroplastic	-1.361
Cg2g025410	Light-harvesting complex II chlorophyll a/b binding protein 6	Chlorophyll a-b binding protein CP24 10B, chloroplastic	-1.234

Cg2g030130			-1.401
Cg2g040770	Photosystem II oxygen-evolving enhancer protein 3	Oxygen-evolving enhancer protein 3, chloroplastic	-2.463
Cg2g044430	Light-harvesting complex II chlorophyll a/b binding protein 1	Chlorophyll a-b binding protein 3C, chloroplastic	-1.333
Cg2g044450	Light-harvesting complex II chlorophyll a/b binding protein 1	Chlorophyll a-b binding protein 3C, chloroplastic	-1.625
Cg4g018890	Mechanosensitive ion channel protein 4/5/6/7/8/9/10		-1.530
Cg5g023620	Light-harvesting complex II chlorophyll a/b binding protein 5	Chlorophyll a-b binding protein CP26, chloroplastic	-1.740
Cg6g006850	Light-harvesting complex II chlorophyll a/b binding protein 4	Chlorophyll a-b binding protein CP29.2, chloroplastic	-1.890
Cg7g005010	Photosystem II oxygen-evolving enhancer protein 2	PsbP-like protein 1, chloroplastic	-1.142
Cg7g006420	Light-harvesting complex I chlorophyll a/b binding protein 1	Chlorophyll a-b binding protein 6A, chloroplastic	-1.626
Cg7g012220	Photosystem II oxygen-evolving enhancer protein 2	Oxygen-evolving enhancer protein 2, chloroplastic	-1.749
Cg7g017970	Photosystem II PsbY protein	Photosystem II core complex proteins psbY, chloroplastic	-1.122
Cg9g006330	Mitochondrial pyruvate carrier 2	Early light-induced protein 1, chloroplastic {ECO:0000305}	1.715
Photosynthetic electron transport chain (GO:0009767)			
Cg4g017260	Photosystem I subunit V	Photosystem I reaction center subunit V, chloroplastic	-1.799
Cg5g001280	2-hydroxyacyl-CoA lyase 1 [EC:4.1.-.-]	NAD(P)H-quinone oxidoreductase subunit S, chloroplastic {ECO:0000305}	-1.221
Cg6g024950	Cell division protease FtsH [EC:3.4.24.-]	ATP-dependent zinc metalloprotease FTSH 6, chloroplastic	2.825
Cg8g013250	Photosystem I subunit PsaO	Photosystem I subunit O	-2.645
Photosystem II oxygen evolving complex (GO:0009654)			
Cg2g030130			-1.401
Cg2g040770	Photosystem II oxygen-evolving enhancer protein 3	Oxygen-evolving enhancer protein 3, chloroplastic	-2.463
Cg7g005010	Photosystem II oxygen-evolving enhancer protein 2	PsbP-like protein 1, chloroplastic	-1.142
Cg7g012220	Photosystem II oxygen-evolving enhancer protein 2	Oxygen-evolving enhancer protein 2, chloroplastic	-1.749
Photosynthesis, light reaction (GO:0019684)			
Cg2g014580	Photosystem II Psb27 protein	Photosystem II repair protein PSB27-H1, chloroplastic	-1.149
Cg2g020050	Light-harvesting complex II chlorophyll a/b binding protein 2	Chlorophyll a-b binding protein 151, chloroplastic	-1.880
Cg2g021790	Light-harvesting complex II chlorophyll a/b binding protein 3	Chlorophyll a-b binding protein 13, chloroplastic	-1.361
Cg2g025410	Light-harvesting complex II chlorophyll a/b binding protein 6	Chlorophyll a-b binding protein CP24 10B, chloroplastic	-1.234
Cg2g044430	Light-harvesting complex II chlorophyll	Chlorophyll a-b binding protein 3C,	-1.333

	a/b binding protein 1	chloroplastic	
Cg2g044450	Light-harvesting complex II chlorophyll a/b binding protein 1	Chlorophyll a-b binding protein 3C, chloroplastic	-1.625
Cg4g017260	Photosystem I subunit V	Photosystem I reaction center subunit V, chloroplastic	-1.799
Cg5g001280	2-hydroxyacyl-CoA lyase 1 [EC:4.1.-.-]	NAD(P)H-quinone oxidoreductase subunit S, chloroplastic {ECO:0000305}	-1.221
Cg5g023620	Light-harvesting complex II chlorophyll a/b binding protein 5	Chlorophyll a-b binding protein CP26, chloroplastic	-1.740
Cg6g006850	Light-harvesting complex II chlorophyll a/b binding protein 4	Chlorophyll a-b binding protein CP29.2, chloroplastic	-1.890
Cg7g006420	Light-harvesting complex I chlorophyll a/b binding protein 1	Chlorophyll a-b binding protein 6A, chloroplastic	-1.626
Cg8g013250	Photosystem I subunit PsaO	Photosystem I subunit O	-2.645
Cg6g024950	Cell division protease FtsH [EC:3.4.24.-]	ATP-dependent zinc metalloprotease FTSH 6, chloroplastic	2.825
Thylakoid (GO:0009579)			
Cg1g024920	Photosystem I subunit PsaN	Photosystem I reaction center subunit N, chloroplastic	-1.265
Cg1g027120	Violaxanthin de-epoxidase [EC:1.23.5.1]	Violaxanthin de-epoxidase, chloroplastic	1.227
Cg2g001710	Enoyl-[acyl-carrier protein] reductase I [EC:1.3.1.9 1.3.1.10]	Enoyl-[acyl-carrier-protein] reductase [NADH], chloroplastic	1.679
Cg2g014580	Photosystem II Psb27 protein	Photosystem II repair protein PSB27-H1, chloroplastic	-1.149
Cg2g020050	Light-harvesting complex II chlorophyll a/b binding protein 2	Chlorophyll a-b binding protein 151, chloroplastic	-1.880
Cg2g021790	Light-harvesting complex II chlorophyll a/b binding protein 3	Chlorophyll a-b binding protein 13, chloroplastic	-1.361
Cg2g025410	Light-harvesting complex II chlorophyll a/b binding protein 6	Chlorophyll a-b binding protein CP24 10B, chloroplastic	-1.234
Cg2g030130			-1.401
Cg2g038220	Photosystem I subunit III	Photosystem I reaction center subunit III, chloroplastic	-1.014
Cg2g040770	Photosystem II oxygen-evolving enhancer protein 3	Oxygen-evolving enhancer protein 3, chloroplastic	-2.463
Cg2g044230	Translation initiation factor 3 subunit D	Cold-regulated 413 inner membrane protein 1, chloroplastic	1.369
Cg2g044430	Light-harvesting complex II chlorophyll a/b binding protein 1	Chlorophyll a-b binding protein 3C, chloroplastic	-1.333
Cg2g044450	Light-harvesting complex II chlorophyll a/b binding protein 1	Chlorophyll a-b binding protein 3C, chloroplastic	-1.625
Cg2g044950	9-cis-epoxycarotenoid dioxygenase [EC:1.13.11.51]	9-cis-epoxycarotenoid dioxygenase NCED1, chloroplastic	-3.481
Cg3g024680	Plastocyanin	Plastocyanin, chloroplastic	-1.448
Cg4g006740	Solute carrier family 39 (zinc transporter), member 1/2/3	Zinc transporter 4, chloroplastic	-1.718
Cg4g017260	Photosystem I subunit V	Photosystem I reaction center subunit V, chloroplastic	-1.799

Cg4g018890	Mechanosensitive ion channel protein 4/5/6/7/8/9/10		-1.530
Cg5g001280	2-hydroxyacyl-CoA lyase 1 [EC:4.1.-.-]	NAD(P)H-quinone oxidoreductase subunit S, chloroplastic {ECO:0000305}	-1.221
Cg5g009510	Magnesium dechelatase [EC:4.99.1.10]	Protein STAY-GREEN 1, chloroplastic	1.601
Cg5g018720	Arsenate reductase [EC:1.20.4.1]	Rhodanese-like domain-containing protein 14, chloroplastic	-1.171
Cg5g023620	Light-harvesting complex II chlorophyll a/b binding protein 5	Chlorophyll a-b binding protein CP26, chloroplastic	-1.740
Cg5g031990	2-methylene-furan-3-one reductase [EC:1.3.1.105]	Chloroplast envelope quinone oxidoreductase homolog {ECO:0000303|PubMed:20424175}	1.736
Cg5g038890		Nitrogen regulatory protein P-II homolog	1.759
Cg6g006850	Light-harvesting complex II chlorophyll a/b binding protein 4	Chlorophyll a-b binding protein CP29.2, chloroplastic	-1.890
Cg6g024950	Cell division protease FtsH [EC:3.4.24.-]	ATP-dependent zinc metalloprotease FTSH 6, chloroplastic	2.825
Cg7g005010	Photosystem II oxygen-evolving enhancer protein 2	PsbP-like protein 1, chloroplastic	-1.142
Cg7g006420	Light-harvesting complex I chlorophyll a/b binding protein 1	Chlorophyll a-b binding protein 6A, chloroplastic	-1.626
Cg7g012220	Photosystem II oxygen-evolving enhancer protein 2	Oxygen-evolving enhancer protein 2, chloroplastic	-1.749
Cg7g012360	Glutathione S-transferase [EC:2.5.1.18]	Glutathione S-transferase F9	-1.318
Cg7g014530	Pyruvate dehydrogenase E2 component (dihydrolipoamide acetyltransferase) [EC:2.3.1.12]	Dihydrolipoyllysine-residue acetyltransferase component 4 of pyruvate dehydrogenase complex, chloroplastic	1.067
Cg7g017970	Photosystem II PsbY protein	Photosystem II core complex proteins psbY, chloroplastic	-1.122
Cg8g000920	Signal recognition particle 43 kDa protein	Signal recognition particle 43 kDa protein, chloroplastic	-1.144
Cg8g013250	Photosystem I subunit PsaO	Photosystem I subunit O	-2.645
Cg9g005290	Photosystem I subunit II	Photosystem I reaction center subunit II, chloroplastic	-1.501
Cg9g006330	Mitochondrial pyruvate carrier 2	Early light-induced protein 1, chloroplastic {ECO:0000305}	1.715
Cg9g006690		Photosynthetic NDH subunit of subcomplex B 5, chloroplastic {ECO:0000303|PubMed:21785130}	-1.012
CgUng002630	ATPase family AAA domain-containing protein 2 [EC:3.6.1.-]	Thylakoid lumenal 15.0 kDa protein 2, chloroplastic	-1.004
	Photosynthesis, dark reaction (GO:0019685)		
Cg4g021720	Ribulose-bisphosphate carboxylase small chain [EC:4.1.1.39]	Ribulose bisphosphate carboxylase small chain, chloroplastic	1.954
Cg7g006810	Transketolase [EC:2.2.1.1]	Transketolase-1, chloroplastic	4.959
	Carbon fixation in photosynthetic organisms (ko00710)		
Cg5g000690	Malate dehydrogenase [EC:1.1.1.37]	Oligopeptide transporter 7	2.225
Cg9g002710	Malate dehydrogenase (decarboxylating) [EC:1.1.1.39]	F-box protein At2g17036	2.633
Cg7g006810	Transketolase [EC:2.2.1.1]	Transketolase-1, chloroplastic	4.959

Cg2g008710	Phosphoenolpyruvate carboxylase [EC:4.1.1.31]	Phosphoenolpyruvate carboxylase 2	1.414
Cg4g021720	Ribulose-bisphosphate carboxylase small chain [EC:4.1.1.39]	Ribulose bisphosphate carboxylase small chain, chloroplastic	1.954
Cg3g013480	Phosphoenolpyruvate carboxykinase (ATP) [EC:4.1.1.49]	Phosphoenolpyruvate carboxykinase (ATP)	3.955

Table S11. DEGs involved in carbon, carbohydrate and energy metabolisms in leaves of *C. grandis* seedlings exposed to Cu-toxicity

Accession No.	KEGG	Swiss-Prot	Log_2(FC)
	Carbon metabolism (ko01200)		
Cg5g000690	Malate dehydrogenase [EC:1.1.1.37]	Oligopeptide transporter 7	2.225
Cg9g002710	Malate dehydrogenase (decarboxylating) [EC:1.1.1.39]	F-box protein At2g17036	2.633
Cg5g000260	Pyruvate dehydrogenase E1 component alpha subunit [EC:1.2.4.1]	Pyruvate dehydrogenase E1 component subunit alpha-3, chloroplastic	2.284
Cg5g040000	Pyruvate dehydrogenase E1 component alpha subunit [EC:1.2.4.1]	Pyruvate dehydrogenase E1 component subunit alpha-3, chloroplastic	1.723
Cg2g044960	Pyruvate dehydrogenase E1 component beta subunit [EC:1.2.4.1]	Pyruvate dehydrogenase E1 component subunit beta-3, chloroplastic	1.708
Cg3g020270	Pyruvate dehydrogenase E2 component (dihydrolipoamide acetyltransferase) [EC:2.3.1.12]	Dihydrolipoyllysine-residue acetyltransferase component 5 of pyruvate dehydrogenase complex, chloroplastic	1.308
Cg7g014530	Pyruvate dehydrogenase E2 component (dihydrolipoamide acetyltransferase) [EC:2.3.1.12]	Dihydrolipoyllysine-residue acetyltransferase component 4 of pyruvate dehydrogenase complex, chloroplastic	1.067
Cg8g002550	Pyruvate kinase [EC:2.7.1.40]	Pyruvate kinase isozyme A, chloroplastic	1.707
Cg9g006500	Pyruvate kinase [EC:2.7.1.40]	Pyruvate kinase isozyme A, chloroplastic	1.238
Cg9g028010	Pyruvate kinase [EC:2.7.1.40]	Plastidial pyruvate kinase 2	1.374
Cg2g008710	Phosphoenolpyruvate carboxylase [EC:4.1.1.31]	Phosphoenolpyruvate carboxylase 2	1.414
Cg3g013480	Phosphoenolpyruvate carboxykinase (ATP) [EC:4.1.1.49]	Phosphoenolpyruvate carboxykinase (ATP)	3.955
Cg3g015300	2,3-bisphosphoglycerate-dependent phosphoglycerate mutase [EC:5.4.2.11]	Metal-independent phosphoserine phosphatase	1.759
Cg4g020570	Acetyl-CoA carboxylase, biotin carboxylase subunit [EC:6.4.1.2 6.3.4.14]	Biotin carboxylase 2, chloroplastic	2.541
Cg5g042320	Acetyl-CoA carboxylase carboxyl transferase subunit alpha [EC:6.4.1.2 2.1.3.15]		1.817
Cg6g025700	Acetyl-CoA carboxylase biotin carboxyl carrier protein	Biotin carboxyl carrier protein of acetyl-CoA carboxylase 1, chloroplastic	1.829
Cg9g001500	Dihydrolipoamide dehydrogenase [EC:1.8.1.4]	Dihydrolipoyl dehydrogenase 1, chloroplastic	1.144
Cg7g006810	Transketolase [EC:2.2.1.1]	Transketolase-1, chloroplastic	4.959
Cg2g020490	Acetyl-CoA C-acetyltransferase [EC:2.3.1.9]	Acetyl-CoA acetyltransferase, cytosolic 1	1.243
Cg5g009780	(S)-2-hydroxy-acid oxidase [EC:1.1.3.15]	Probable transcriptional regulator SLK2	2.193
Cg5g007370	Glutamate dehydrogenase (NAD(P)$^+$) [EC:1.4.1.3]	Glutamate dehydrogenase 2	1.745
Cg4g021720	Ribulose-bisphosphate carboxylase small chain [EC:4.1.1.39]	Ribulose bisphosphate carboxylase small chain, chloroplastic	1.954
Cg4g004610	6-phosphofructokinase 1 [EC:2.7.1.11]	ATP-dependent 6-phosphofructokinase 3	1.958
Cg1g013660	6-phosphofructokinase 1 [EC:2.7.1.11]	ATP-dependent 6-phosphofructokinase 3	1.420
Cg7g022330	6-phosphofructokinase 1 [EC:2.7.1.11]	Acyl carrier protein 1, chloroplastic	-1.663
Cg7g016170	6-phosphogluconolactonase [EC:3.1.1.31]	Probable 6-phosphogluconolactonase 1	-1.087
Cg9g001560	Acetate/butyrate---CoA ligase [EC:6.2.1.1	Acetate/butyrate--CoA ligase AAE7,	-1.161

	6.2.1.2]	peroxisomal	
Cg6g007590	S-(hydroxymethyl)glutathione dehydrogenase / alcohol dehydrogenase [EC:1.1.1.284 1.1.1.1]	Alcohol dehydrogenase-like 2	-2.347
Cg5g008070	3-hydroxyisobutyryl-CoA hydrolase [EC:3.1.2.4]	3-hydroxyisobutyryl-CoA hydrolase-like protein 2, mitochondrial	-1.318
Cg3g025260	Catalase [EC:1.11.1.6]	Catalase	-1.191
	Starch and sucrose metabolism (ko00500)		
Cg4g018060	Sucrose synthase [EC:2.4.1.13]	Sucrose synthase	1.848
Cg9g002460	Sucrose synthase [EC:2.4.1.13]	Sucrose synthase 2	3.483
Cg4g002030	Sucrose-phosphate synthase [EC:2.4.1.14]	Probable sucrose-phosphate synthase 1	1.365
Cg3g025200	1,4-alpha-glucan branching enzyme [EC:2.4.1.18]	1,4-alpha-glucan-branching enzyme 1, chloroplastic/amyloplastic	1.472
Cg6g016110	1,4-alpha-glucan branching enzyme [EC:2.4.1.18]	1,4-alpha-glucan-branching enzyme 1, chloroplastic/amyloplastic	1.133
Cg4g020710	Isoamylase [EC:3.2.1.68]	Isoamylase 1, chloroplastic	1.749
Cg5g008610	Trehalose 6-phosphate phosphatase [EC:3.1.3.12]	Probable trehalose-phosphate phosphatase G	1.209
Cg2g006510	Trehalose 6-phosphate phosphatase [EC:3.1.3.12]	Protein KINESIN LIGHT CHAIN-RELATED 2	-1.191
Cg4g022560	Trehalose 6-phosphate synthase/phosphatase [EC:2.4.1.15 3.1.3.12]	Probable alpha,alpha-trehalose-phosphate synthase [UDP-forming] 11	1.050
Cg5g004730	Trehalose 6-phosphate synthase/phosphatase [EC:2.4.1.15 3.1.3.12]	Probable alpha,alpha-trehalose-phosphate synthase [UDP-forming] 9	-2.681
Cg3g024770	Alpha-amylase [EC:3.2.1.1]	Probable alpha-amylase 2	1.243
Cg3g021390	Alpha-amylase [EC:3.2.1.1]	Alpha-amylase	-2.293
Cg2g017760	Beta-amylase [EC:3.2.1.2]	Beta-amylase	-1.926
Cg5g008630	Beta-fructofuranosidase [EC:3.2.1.26]	Acid beta-fructofuranosidase	3.237
Cg5g037320	Beta-glucosidase [EC:3.2.1.21]	Beta-glucosidase 11	1.166
Cg6g000800	Beta-glucosidase [EC:3.2.1.21]	Subtilisin-like protease SBT1.9	2.274
Cg8g014060	Glucan endo-1,3-beta-glucosidase 1/2/3 [EC:3.2.1.39]	Glucan endo-1,3-beta-glucosidase 14	1.055
Cg8g022430	Glucan endo-1,3-beta-glucosidase 1/2/3 [EC:3.2.1.39]	Glucan endo-1,3-beta-glucosidase	1.465
Cg9g018300	Glucan endo-1,3-beta-glucosidase 1/2/3 [EC:3.2.1.39]	Glucan endo-1,3-beta-glucosidase, basic isoform	-2.343
Cg3g023860	Endoglucanase [EC:3.2.1.4]	Endoglucanase 10	1.121
	Glycolysis / gluconeogenesis (ko00010)		
Cg6g005300	Alcohol dehydrogenase ($NADP^+$) [EC:1.1.1.2]	NADPH-dependent aldo-keto reductase, chloroplastic	1.372
Cg7g019540	Aldehyde dehydrogenase (NAD^+) [EC:1.2.1.3]	Aldehyde dehydrogenase family 2 member B7, mitochondrial	-1.801
Cg5g032800	Alcohol dehydrogenase class-P [EC:1.1.1.1]	Alcohol dehydrogenase	-1.629
Cg5g000260	Pyruvate dehydrogenase E1 component alpha subunit [EC:1.2.4.1]	Pyruvate dehydrogenase E1 component subunit alpha-3, chloroplastic	2.284
Cg5g040000	Pyruvate dehydrogenase E1 component alpha subunit [EC:1.2.4.1]	Pyruvate dehydrogenase E1 component subunit alpha-3, chloroplastic	1.723
Cg2g044960	Pyruvate dehydrogenase E1 component	Pyruvate dehydrogenase E1 component	1.708

	beta subunit [EC:1.2.4.1]	subunit beta-3, chloroplastic	
Cg3g020270	Pyruvate dehydrogenase E2 component (dihydrolipoamide acetyltransferase) [EC:2.3.1.12]	Dihydrolipoyllysine-residue acetyltransferase component 5 of pyruvate dehydrogenase complex, chloroplastic	1.308
Cg7g014530	Pyruvate dehydrogenase E2 component (dihydrolipoamide acetyltransferase) [EC:2.3.1.12]	Dihydrolipoyllysine-residue acetyltransferase component 4 of pyruvate dehydrogenase complex, chloroplastic	1.067
Cg8g002550	Pyruvate kinase [EC:2.7.1.40]	Pyruvate kinase isozyme A, chloroplastic	1.707
Cg9g006500	Pyruvate kinase [EC:2.7.1.40]	Pyruvate kinase isozyme A, chloroplastic	1.238
Cg9g028010	Pyruvate kinase [EC:2.7.1.40]	Plastidial pyruvate kinase 2	1.374
Cg6g009400	Pyruvate decarboxylase [EC:4.1.1.1]	Pyruvate decarboxylase 1	1.236
Cg3g013480	Phosphoenolpyruvate carboxykinase (ATP) [EC:4.1.1.49]	Phosphoenolpyruvate carboxykinase (ATP)	3.955
Cg9g001500	Dihydrolipoamide dehydrogenase [EC:1.8.1.4]	Dihydrolipoyl dehydrogenase 1, chloroplastic	1.144
Cg5g041140	Glucose-6-phosphate 1-epimerase [EC:5.1.3.15]	Putative glucose-6-phosphate 1-epimerase	1.298
Cg3g015300	2,3-bisphosphoglycerate-dependent phosphoglycerate mutase [EC:5.4.2.11]	Metal-independent phosphoserine phosphatase	1.759
Cg4g004610	6-phosphofructokinase 1 [EC:2.7.1.11]	ATP-dependent 6-phosphofructokinase 3	1.958
Cg1g013660	6-phosphofructokinase 1 [EC:2.7.1.11]	ATP-dependent 6-phosphofructokinase 3	1.420
Cg7g022330	6-phosphofructokinase 1 [EC:2.7.1.11]	Acyl carrier protein 1, chloroplastic	-1.663
Cg6g018080	Aldose 1-epimerase [EC:5.1.3.3]		-3.506
Cg9g001560	Acetate/butyrate---CoA ligase [EC:6.2.1.1 6.2.1.2]	Acetate/butyrate--CoA ligase AAE7, peroxisomal	-1.161
Cg6g007590	S-(hydroxymethyl)glutathione dehydrogenase / alcohol dehydrogenase [EC:1.1.1.284 1.1.1.1]	Alcohol dehydrogenase-like 2	-2.347
	Glycolytic process (GO:0006096)		
Cg2g044960	Pyruvate dehydrogenase E1 component beta subunit [EC:1.2.4.1]	Pyruvate dehydrogenase E1 component subunit beta-3, chloroplastic	1.708
Cg3g005210		Ethylene-responsive transcription factor WRI1	2.575
Cg3g020270	Pyruvate dehydrogenase E2 component (dihydrolipoamide acetyltransferase) [EC:2.3.1.12]	Dihydrolipoyllysine-residue acetyltransferase component 5 of pyruvate dehydrogenase complex, chloroplastic	1.308
Cg5g000260	Pyruvate dehydrogenase E1 component alpha subunit [EC:1.2.4.1]	Pyruvate dehydrogenase E1 component subunit alpha-3, chloroplastic	2.284
Cg5g040000	Pyruvate dehydrogenase E1 component alpha subunit [EC:1.2.4.1]	Pyruvate dehydrogenase E1 component subunit alpha-3, chloroplastic	1.723
Cg7g014530	Pyruvate dehydrogenase E2 component (dihydrolipoamide acetyltransferase) [EC:2.3.1.12]	Dihydrolipoyllysine-residue acetyltransferase component 4 of pyruvate dehydrogenase complex, chloroplastic	1.067
Cg1g013660	6-phosphofructokinase 1 [EC:2.7.1.11]	ATP-dependent 6-phosphofructokinase 3	1.420
Cg4g004610	6-phosphofructokinase 1 [EC:2.7.1.11]	ATP-dependent 6-phosphofructokinase 3	1.958
	Pyruvate metabolism (ko00620)		
Cg5g000690	Malate dehydrogenase [EC:1.1.1.37]	Oligopeptide transporter 7	2.225
Cg9g002710	Malate dehydrogenase (decarboxylating) [EC:1.1.1.39]	F-box protein At2g17036	2.633
Cg5g000260	Pyruvate dehydrogenase E1 component	Pyruvate dehydrogenase E1 component	2.284

	alpha subunit [EC:1.2.4.1]	subunit alpha-3, chloroplastic	
Cg5g040000	Pyruvate dehydrogenase E1 component alpha subunit [EC:1.2.4.1]	Pyruvate dehydrogenase E1 component subunit alpha-3, chloroplastic	1.723
Cg2g044960	Pyruvate dehydrogenase E1 component beta subunit [EC:1.2.4.1]	Pyruvate dehydrogenase E1 component subunit beta-3, chloroplastic	1.708
Cg3g020270	Pyruvate dehydrogenase E2 component (dihydrolipoamide acetyltransferase) [EC:2.3.1.12]	Dihydrolipoyllysine-residue acetyltransferase component 5 of pyruvate dehydrogenase complex, chloroplastic	1.308
Cg7g014530	Pyruvate dehydrogenase E2 component (dihydrolipoamide acetyltransferase) [EC:2.3.1.12]	Dihydrolipoyllysine-residue acetyltransferase component 4 of pyruvate dehydrogenase complex, chloroplastic	1.067
Cg8g002550	Pyruvate kinase [EC:2.7.1.40]; Pyruvate kinase isozyme A, chloroplastic		1.707
Cg9g006500	Pyruvate kinase [EC:2.7.1.40]	Pyruvate kinase isozyme A, chloroplastic	1.238
Cg9g028010	Pyruvate kinase [EC:2.7.1.40]	Plastidial pyruvate kinase 2	1.374
Cg2g008710	Phosphoenolpyruvate carboxylase [EC:4.1.1.31]	Phosphoenolpyruvate carboxylase 2	1.414
Cg3g013480	Phosphoenolpyruvate carboxykinase (ATP) [EC:4.1.1.49]	Phosphoenolpyruvate carboxykinase (ATP)	3.955
Cg9g001500	Dihydrolipoamide dehydrogenase [EC:1.8.1.4]	Dihydrolipoyl dehydrogenase 1, chloroplastic	1.144
Cg2g020490	Acetyl-CoA C-acetyltransferase [EC:2.3.1.9]	Acetyl-CoA acetyltransferase, cytosolic 1	1.243
Cg4g020570	Acetyl-CoA carboxylase, biotin carboxylase subunit [EC:6.4.1.2 6.3.4.14]	Biotin carboxylase 2, chloroplastic	2.541
Cg5g042320	Acetyl-CoA carboxylase carboxyl transferase subunit alpha [EC:6.4.1.2 2.1.3.15]		1.817
Cg6g025700	Acetyl-CoA carboxylase biotin carboxyl carrier protein	Biotin carboxyl carrier protein of acetyl-CoA carboxylase 1, chloroplastic	1.829
Cg9g001560	Acetate/butyrate---CoA ligase [EC:6.2.1.1 6.2.1.2]	Acetate/butyrate--CoA ligase AAE7, peroxisomal	-1.161
Cg7g019540	Aldehyde dehydrogenase (NAD^+) [EC:1.2.1.3]	Aldehyde dehydrogenase family 2 member B7, mitochondrial	-1.801
Citrate cycle (TCA cycle) (ko00020)			
Cg5g000690	Malate dehydrogenase [EC:1.1.1.37]	Oligopeptide transporter 7	2.225
Cg5g000260	Pyruvate dehydrogenase E1 component alpha subunit [EC:1.2.4.1]	Pyruvate dehydrogenase E1 component subunit alpha-3, chloroplastic	2.284
Cg5g040000	Pyruvate dehydrogenase E1 component alpha subunit [EC:1.2.4.1]	Pyruvate dehydrogenase E1 component subunit alpha-3, chloroplastic	1.723
Cg2g044960	Pyruvate dehydrogenase E1 component beta subunit [EC:1.2.4.1]	Pyruvate dehydrogenase E1 component subunit beta-3, chloroplastic	1.708
Cg9g001500	Dihydrolipoamide dehydrogenase [EC:1.8.1.4]	Dihydrolipoyl dehydrogenase 1, chloroplastic	1.144
Cg3g020270	Pyruvate dehydrogenase E2 component (dihydrolipoamide acetyltransferase) [EC:2.3.1.12]	Dihydrolipoyllysine-residue acetyltransferase component 5 of pyruvate dehydrogenase complex, chloroplastic	1.308
Cg7g014530	Pyruvate dehydrogenase E2 component (dihydrolipoamide acetyltransferase)	Dihydrolipoyllysine-residue acetyltransferase component 4 of pyruvate dehydrogenase	1.067

	[EC:2.3.1.12]	complex, chloroplastic	
Cg3g013480	Phosphoenolpyruvate carboxykinase (ATP) [EC:4.1.1.49]	Phosphoenolpyruvate carboxykinase (ATP)	3.955
ATP biosynthetic process (GO:0006754)			
Cg1g013660	6-phosphofructokinase 1 [EC:2.7.1.11]	ATP-dependent 6-phosphofructokinase 3	1.420
Cg2g044960	Pyruvate dehydrogenase E1 component beta subunit [EC:1.2.4.1]	Pyruvate dehydrogenase E1 component subunit beta-3, chloroplastic	1.708
Cg3g020270	Pyruvate dehydrogenase E2 component (dihydrolipoamide acetyltransferase) [EC:2.3.1.12]	Dihydrolipoyllysine-residue acetyltransferase component 5 of pyruvate dehydrogenase complex, chloroplastic	1.308
Cg4g004610	6-phosphofructokinase 1 [EC:2.7.1.11]	ATP-dependent 6-phosphofructokinase 3	1.958
Cg5g000260	Pyruvate dehydrogenase E1 component alpha subunit [EC:1.2.4.1]	Pyruvate dehydrogenase E1 component subunit alpha-3, chloroplastic	2.284
Cg5g040000	Pyruvate dehydrogenase E1 component alpha subunit [EC:1.2.4.1]	Pyruvate dehydrogenase E1 component subunit alpha-3, chloroplastic	1.723
Cg7g014530	Pyruvate dehydrogenase E2 component (dihydrolipoamide acetyltransferase) [EC:2.3.1.12]	Dihydrolipoyllysine-residue acetyltransferase component 4 of pyruvate dehydrogenase complex, chloroplastic	1.067
Cg3g005210		Ethylene-responsive transcription factor WRI1	2.575
Cg9g013190	F-type H^+-transporting ATPase subunit b	Putative ATP synthase protein YMF19	-1.145
Cg1g013420	H^+-transporting ATPase [EC:3.6.3.6]	ATPase 10, plasma membrane-type	-3.022
	ATP generation from ADP (GO:0006757)		
Cg1g013660	6-phosphofructokinase 1 [EC:2.7.1.11]	ATP-dependent 6-phosphofructokinase 3	1.420
Cg2g044960	Pyruvate dehydrogenase E1 component beta subunit [EC:1.2.4.1]	Pyruvate dehydrogenase E1 component subunit beta-3, chloroplastic	1.708
Cg3g020270	Pyruvate dehydrogenase E2 component (dihydrolipoamide acetyltransferase) [EC:2.3.1.12]	Dihydrolipoyllysine-residue acetyltransferase component 5 of pyruvate dehydrogenase complex, chloroplastic	1.308
Cg4g004610	6-phosphofructokinase 1 [EC:2.7.1.11]	ATP-dependent 6-phosphofructokinase 3	1.958
Cg5g000260	Pyruvate dehydrogenase E1 component alpha subunit [EC:1.2.4.1]	Pyruvate dehydrogenase E1 component subunit alpha-3, chloroplastic	2.284
Cg5g040000	Pyruvate dehydrogenase E1 component alpha subunit [EC:1.2.4.1]	Pyruvate dehydrogenase E1 component subunit alpha-3, chloroplastic	1.723
Cg7g014530	Pyruvate dehydrogenase E2 component (dihydrolipoamide acetyltransferase) [EC:2.3.1.12]	Dihydrolipoyllysine-residue acetyltransferase component 4 of pyruvate dehydrogenase complex, chloroplastic	1.067
Cg3g005210		Ethylene-responsive transcription factor WRI1	2.575

Table S12. DEGs involved in photorespiration (GO:0009853), glutathione metabolism (ko00480), cell redox homeostasis (GO:0045454) and others involved in thermal dissipation and ROS scavenging in leaves of *C. grandis* seedlings exposed to Cu-toxicity

Accession No.	KEGG	Swiss-Prot	Log_2(FC)
	Photorespiration (GO:0009853)		
Cg4g009780	Alanine-glyoxylate transaminase / (R)-3-amino-2-methylpropionate-pyruvate transaminase [EC:2.6.1.44 2.6.1.40]	Alanine--glyoxylate aminotransferase 2 homolog 2, mitochondrial	1.357
Cg6g010200	Alanine-glyoxylate transaminase / (R)-3-amino-2-methylpropionate-pyruvate transaminase [EC:2.6.1.44 2.6.1.40]	Alanine--glyoxylate aminotransferase 2 homolog 3, mitochondrial	1.702
Cg4g021720	Ribulose-bisphosphate carboxylase small chain [EC:4.1.1.39]	Ribulose bisphosphate carboxylase small chain, chloroplastic	1.954
	Glutathione metabolism (ko00480)		
Cg5g026120	Ornithine decarboxylase [EC:4.1.1.17]	Ornithine decarboxylase	-1.980
Cg6g003160	Glutathione S-transferase [EC:2.5.1.18]	Glutathione S-transferase L3	-1.072
Cg7g012360	Glutathione S-transferase [EC:2.5.1.18]	Glutathione S-transferase F9	-1.318
Cg8g023290	Glutathione S-transferase [EC:2.5.1.18]	Glutathione S-transferase U7	-1.268
Cg9g008200	Glutathione S-transferase [EC:2.5.1.18]	Probable glutathione S-transferase	-1.889
Cg9g008210	Glutathione S-transferase [EC:2.5.1.18]	Probable glutathione S-transferase	-2.100
Cg9g008220	Glutathione S-transferase [EC:2.5.1.18]	Probable glutathione S-transferase	-1.754
Cg2g030200	Glutathione S-transferase [EC:2.5.1.18]	Cyclin-P3-1	-2.011
Cg8g023310	Glutathione S-transferase [EC:2.5.1.18]	Probable glutathione S-transferase	2.050
Cg6g002810	L-ascorbate peroxidase [EC:1.11.1.11]	L-ascorbate peroxidase 2, cytosolic	1.880
Cg2g031080	Leucyl aminopeptidase [EC:3.4.11.1]		1.399
Cg5g017120	Gamma-glutamyltranspeptidase / glutathione hydrolase / leukotriene-C4 hydrolase [EC:2.3.2.2 3.4.19.13 3.4.19.14];	Glutathione hydrolase 3	1.155
Cg2g023200	Cell redox homeostasis (GO:0045454)		
	Thioredoxin 1	Thioredoxin-like protein CXXS1	-2.322
Cg9g003330	Glutaredoxin 3	Monothiol glutaredoxin-S10	-5.294
Cg9g003340	Glutaredoxin 3	Monothiol glutaredoxin-S2	-2.265
Cg2g042710	Glutaredoxin 3	Glutaredoxin-C9	3.564
Cg3g022900		CBS domain-containing protein CBSX3, mitochondrial	1.571
Cg3g024580	Nucleoredoxin [EC:1.8.1.8]	Probable nucleoredoxin 2	1.855
Cg5g036730	Glutaredoxin 3	Monothiol glutaredoxin-S9	2.627
Cg9g001500	Dihydrolipoamide dehydrogenase [EC:1.8.1.4]	Dihydrolipoyl dehydrogenase 1, chloroplastic	1.144
	Others		
Cg1g027120	Violaxanthin de-epoxidase [EC:1.23.5.1]	Violaxanthin de-epoxidase, chloroplastic	1.227
Cg7g011780	Superoxide dismutase, Fe-Mn family [EC:1.15.1.1]	Superoxide dismutase [Fe], chloroplastic	-2.052
Cg8g018870	Superoxide dismutase, Cu-Zn family [EC:1.15.1.1]	Superoxide dismutase [Cu-Zn], chloroplastic	1.344
Cg5g009340	Copper chaperone for superoxide dismutase	Copper chaperone for superoxide dismutase, chloroplastic/cytosolic; AtCCS	1.588
Cg3g025260	Catalase [EC:1.11.1.6]	Catalase	-1.191

Cg1g002980	Peroxidase [EC:1.11.1.7]	Peroxidase 10	5.291
Cg1g008060	Peroxidase [EC:1.11.1.7]	Peroxidase 4	1.890
Cg2g001370	Peroxidase [EC:1.11.1.7]	Peroxidase 15	-1.607
Cg2g001400	Peroxidase [EC:1.11.1.7]	Peroxidase 15	-2.280
Cg2g001440	Peroxidase [EC:1.11.1.7]	Peroxidase 15 {ECO:0000303|PubMed:17936696}	-3.086
Cg2g001460	Peroxidase [EC:1.11.1.7]	Peroxidase C3	-4.173
Cg3g005370	Peroxidase [EC:1.11.1.7]	Peroxidase 15 {ECO:0000303|PubMed:17936696}	-2.085

Table S13. DEGs involved in Ca homeostasis in leaves of *C. grandis* seedlings exposed to Cu-toxicity

Accession No.	KEGG	Swiss-Prot	Log_2(FC)
	Calcium ion transmembrane transporter activity (GO:0015085)		
Cg5g012700	Ca^{2+}-transporting ATPase [EC:3.6.3.8]	Calcium-transporting ATPase 9, plasma membrane-type	-1.120
Cg2g020060	Ca^{2+}-transporting ATPase [EC:3.6.3.8]	Calcium-transporting ATPase 12, plasma membrane-type	-1.457
Cg5g034830	Ca^{2+}-transporting ATPase [EC:3.6.3.8]	Calcium-transporting ATPase 12, plasma membrane-type	-1.593
Cg3g010620	Ca^{2+}-transporting ATPase [EC:3.6.3.8]	Calcium-transporting ATPase 12, plasma membrane-type	2.368
Cg6g003500	Ca^{2+}-transporting ATPase [EC:3.6.3.8]	Calcium-transporting ATPase 4, endoplasmic reticulum-type	1.274
Cg3g022310	Calcium uniporter protein, mitochondrial	Calcium uniporter protein 2, mitochondrial	2.306
Cg4g022490	Glutamate receptor, ionotropic, plant	Glutamate receptor 2.8; Ligand-gated ion channel 2.8	2.004
Cg6g006900	Ca^{2+}:H^{+} antiporter	Vacuolar cation/proton exchanger 3; Ca(2+)/H(+) antiporter CAX3; Ca(2+)/H(+) exchanger 3; Protein CATION EXCHANGER 3;	-1.317
Cg8g024060	Ca^{2+}:H^{+} antiporter	Vacuolar cation/proton exchanger 3; $Ca(^{2+})/H(^{+})$ antiporter CAX3; Ca(2+)/H(+) exchanger 3; Protein CATION EXCHANGER 3;	-1.613
	Calcium-mediated signaling (GO:0019722)		
Cg4g022490	Glutamate receptor, ionotropic, plant	Glutamate receptor 2.8; Ligand-gated ion channel 2.8	2.004
Cg9g014450		Protein RALF-like 32	-1.347
	Calmodulin binding (GO:0005516)		
Cg2g020060	Ca^{2+}-transporting ATPase [EC:3.6.3.8]	Calcium-transporting ATPase 12, plasma membrane-type	-1.457
Cg3g010620	Ca^{2+}-transporting ATPase [EC:3.6.3.8]	Calcium-transporting ATPase 12, plasma membrane-type	2.368
Cg5g012700	Ca^{2+}-transporting ATPase [EC:3.6.3.8]	Calcium-transporting ATPase 9, plasma membrane-type	-1.120
Cg5g034830	Ca^{2+}-transporting ATPase [EC:3.6.3.8]	Calcium-transporting ATPase 12, plasma membrane-type	-1.593
Cg2g014880	Interleukin-1 receptor-associated kinase 4 [EC:2.7.11.1]	G-type lectin S-receptor-like serine/threonine-protein kinase At4g03230	-2.095
Cg3g012090	Interleukin-1 receptor-associated kinase 4 [EC:2.7.11.1]	G-type lectin S-receptor-like serine/threonine-protein kinase B120	-3.348
Cg4g002250	Interleukin-1 receptor-associated kinase 4 [EC:2.7.11.1]	G-type lectin S-receptor-like serine/threonine-protein kinase SD2-5	-1.361
Cg8g018400	Interleukin-1 receptor-associated kinase 4 [EC:2.7.11.1]	G-type lectin S-receptor-like serine/threonine-protein kinase At4g03230	-2.581
Cg8g018410	Chitinase [EC:3.2.1.14]	G-type lectin S-receptor-like serine/threonine-protein kinase CES101	-2.087
Cg8g018470	Chitinase [EC:3.2.1.14]	G-type lectin S-receptor-like serine/threonine-	-1.937

		protein kinase At1g11330	
Cg8g010170	Calcium/calmodulin-dependent protein kinase (CaM kinase) II [EC:2.7.11.17]	Phosphoenolpyruvate carboxylase kinase 1	2.727
Cg2g043490	Protein brassinosteroid insensitive 1 [EC:2.7.10.1 2.7.11.1]	Calmodulin-binding receptor kinase CaMRLK {ECO:0000305}	1.303
Cg3g013020	FK506-binding protein 4/5 [EC:5.2.1.8]	Peptidyl-prolyl cis-trans isomerase FKBP62	-7.401
Cg5g006140	E1A/CREB-binding protein [EC:2.3.1.48]	BTB/POZ and TAZ domain-containing protein 2	-1.821
Cg5g018340		Protein IQ-DOMAIN 1 {ECO:0000303|PubMed:15960618}	-2.010
Cg9g027140	WW domain-containing oxidoreductase	Short-chain dehydrogenase TIC 32, chloroplastic	1.256
Cg9g029620	YTH domain-containing family protein	30-kDa cleavage and polyadenylation specificity factor 30 {ECO:0000303|PubMed:18479511}	1.074
	Calmodulin-dependent protein kinase activity (GO:0004683)		
Cg8g010170	Calcium/calmodulin-dependent protein kinase (CaM kinase) II [EC:2.7.11.17]	Phosphoenolpyruvate carboxylase kinase 1	2.727
	Calcium ion binding (GO:0005509)		
Cg1g003570	Calcium-binding protein CML	Probable calcium-binding protein CML41; Calmodulin-like protein 41	-2.486
Cg2g011280	Calcium-binding protein CML	Calcium-binding protein CML38; Calmodulin-like protein 38	-1.707
Cg3g017200	Calcium-binding protein CML	Probable calcium-binding protein CML44; Calmodulin-like protein 44	-1.082
Cg6g011100	Calcium-binding protein CML	Calcium-binding allergen Bet v 3; Allergen Bet v III;	-1.061
Cg3g021480	Calmodulin	Calmodulin-like protein 5	-2.843
Cg5g018200	Serine/threonine-protein phosphatase 2B regulatory subunit | (RefSeq) calcineurin B-like protein 7 (A)	Calcineurin B-like protein 7	-1.649
Cg2g022120	Centrin-1	Calcium-binding protein PBP1; KIC-related protein 2; PINOID-binding protein 1	-1.020
Cg1g015970	Interleukin-1 receptor-associated kinase 4 [EC:2.7.11.1]	Wall-associated receptor kinase-like 10	2.024
Cg5g026300	Interleukin-1 receptor-associated kinase 4 [EC:2.7.11.1]	Wall-associated receptor kinase 5	-3.253
Cg2g024270	Interleukin-1 receptor-associated kinase 4 [EC:2.7.11.1]	Wall-associated receptor kinase-like 2	-1.330
Cg7g005010	Photosystem II oxygen-evolving enhancer protein 2	PsbP-like protein 1, chloroplastic	-1.142
Cg7g012220	Photosystem II oxygen-evolving enhancer protein 2	Oxygen-evolving enhancer protein 2, chloroplastic	-1.749
Cg2g040770	Photosystem II oxygen-evolving enhancer protein 3	Oxygen-evolving enhancer protein 3, chloroplastic	-2.463
Cg3g021390	Alpha-amylase [EC:3.2.1.1]	Alpha-amylase	-2.293
Cg3g024770	Alpha-amylase [EC:3.2.1.1]	Probable alpha-amylase 2	1.243
Cg3g011670			1.974
Cg2g030130			-1.401
Cg3g022310	Calcium channel activity (GO:0005262)		

	Calcium uniporter protein, mitochondrial	Calcium uniporter protein 2, mitochondrial	2.306
Cg4g022490	Glutamate receptor, ionotropic, plant	Glutamate receptor 2.8; Ligand-gated ion channel 2.8	2.004

Table S14. DEGs involved in MAPK signaling pathway - plant (ko04016) in leaves of *C. grandis* seedlings exposed to Cu-toxicity

Accession No.	KEGG	Swiss-Prot	Log_2(FC)
Cg3g014360	Calmodulin	Rhodanese-like domain-containing protein 6	1.692
Cg6g017010	Calmodulin	Rhodanese-like domain-containing protein 6	1.900
Cg3g021480	Calmodulin	Calmodulin-like protein 5	-2.843
Cg3g025260	Catalase [EC:1.11.1.6]	Catalase	-1.191
Cg2g038570	Mitogen-activated protein kinase kinase 4/5 [EC:2.7.12.2]	Protein LIGHT-DEPENDENT SHORT HYPOCOTYLS 6	1.067
Cg9g020900	LRR receptor-like serine/threonine-protein kinase FLS2 [EC:2.7.11.1]	Receptor kinase-like protein Xa21	1.870
Cg4g008420	LRR receptor-like serine/threonine-protein kinase FLS2 [EC:2.7.11.1]	Leucine-rich repeat extensin-like protein 4	-1.176
Cg7g009010	LRR receptor-like serine/threonine-protein kinase FLS2 [EC:2.7.11.1]	Receptor kinase-like protein Xa21	-1.796
Cg9g006130	LRR receptor-like serine/threonine-protein kinase FLS2 [EC:2.7.11.1]	Leucine-rich repeat extensin-like protein 6	-2.603
Cg9g021300	LRR receptor-like serine/threonine-protein kinase FLS2 [EC:2.7.11.1]	Probable LRR receptor-like serine/threonine-protein kinase At3g47570	-1.046
Cg9g021310	LRR receptor-like serine/threonine-protein kinase FLS2 [EC:2.7.11.1]	Probable LRR receptor-like serine/threonine-protein kinase At3g47570	-1.066
Cg9g021330	LRR receptor-like serine/threonine-protein kinase FLS2 [EC:2.7.11.1]	Probable LRR receptor-like serine/threonine-protein kinase At3g47570	-1.084
Cg9g021360	LRR receptor-like serine/threonine-protein kinase FLS2 [EC:2.7.11.1]	Probable LRR receptor-like serine/threonine-protein kinase At3g47570	-1.128
Cg9g021390	LRR receptor-like serine/threonine-protein kinase FLS2 [EC:2.7.11.1]	Probable LRR receptor-like serine/threonine-protein kinase At3g47570	-1.131
CgUng003130	LRR receptor-like serine/threonine-protein kinase FLS2 [EC:2.7.11.1]	LRR receptor-like serine/threonine-protein kinase EFR	-4.316
Cg1g015500	LRR receptor-like serine/threonine-protein kinase FLS2 [EC:2.7.11.1]	LRR receptor-like serine/threonine-protein kinase FLS2	-1.275
Cg1g015520	LRR receptor-like serine/threonine-protein kinase FLS2 [EC:2.7.11.1]	LRR receptor-like serine/threonine-protein kinase GSO2	-1.602
Cg1g015540	LRR receptor-like serine/threonine-protein kinase FLS2 [EC:2.7.11.1]	Probable LRR receptor-like serine/threonine-protein kinase At3g47570	-2.467
Cg1g015570	LRR receptor-like serine/threonine-protein kinase FLS2 [EC:2.7.11.1]	Probable LRR receptor-like serine/threonine-protein kinase At3g47570	-2.068
Cg1g015670	LRR receptor-like serine/threonine-protein kinase FLS2 [EC:2.7.11.1]	Probable LRR receptor-like serine/threonine-protein kinase At3g47570	-1.948
Cg1g015690	LRR receptor-like serine/threonine-protein kinase FLS2 [EC:2.7.11.1]	Receptor kinase-like protein Xa21	-1.414
Cg1g015720	LRR receptor-like serine/threonine-protein kinase FLS2 [EC:2.7.11.1]	Probable LRR receptor-like serine/threonine-protein kinase At3g47570	-2.644
Cg1g020830	LRR receptor-like serine/threonine-protein kinase FLS2 [EC:2.7.11.1]	Receptor like protein 8	-2.458
Cg2g023840	LRR receptor-like serine/threonine-protein kinase FLS2 [EC:2.7.11.1]	Receptor-like protein 12	-1.593
Cg3g001850	LRR receptor-like serine/threonine-	Probable LRR receptor-like serine/threonine-	-1.535

	protein kinase FLS2 [EC:2.7.11.1]	protein kinase At3g47570	
Cg6g012270	LRR receptor-like serine/threonine-protein kinase ERECTA [EC:2.7.11.1]	DNA damage-repair/toleration protein DRT100	-1.908
Cg5g001390	Protein phosphatase 2C [EC:3.1.3.16]	Probable protein phosphatase 2C 8	3.181
Cg7g000680	Protein phosphatase 2C [EC:3.1.3.16]	Protein phosphatase 2C 37	1.252
Cg8g023080	Protein phosphatase 2C [EC:3.1.3.16]	Protein phosphatase 2C 3	2.730
Cg9g028610	Protein phosphatase 2C [EC:3.1.3.16]	Probable protein phosphatase 2C 8	1.958
CgUng003480	Protein phosphatase 2C [EC:3.1.3.16]	Protein phosphatase 2C 56	1.448
Cg1g007240	Transcription factor MYC2	Transcription factor bHLH25	8.612
Cg2g040740	Transcription factor MYC2	Transcription factor ILR3	-1.273
Cg1g008410	Pathogenesis-related protein 1	Basic form of pathogenesis-related protein 1	7.160
Cg6g009750	Abscisic acid receptor PYR/PYL family	Abscisic acid receptor PYL4	-2.059
Cg7g002520	Abscisic acid receptor PYR/PYL family	Abscisic acid receptor PYL4	-2.222
Cg9g008980	Ethylene-insensitive protein 3	Phosphate transporter PHO1	1.909
Cg4g007230	EIN3-binding F-box protein	EIN3-binding F-box protein 1	-1.226
Cg1g000850	Basic endochitinase B [EC:3.2.1.14]	Basic endochitinase	-3.753
Cg8g000790	Basic endochitinase B [EC:3.2.1.14]	Endochitinase	-4.533
Cg8g000800	Basic endochitinase B [EC:3.2.1.14]	Endochitinase	-4.480

Table S15. DEGs involved in plant hormone signal transduction (ko04075) in leaves of *C. grandis* seedlings exposed to Cu-toxicity

Accession No.	KEGG	Swiss-Prot	Log_2(FC)
	Auxin		
Cg6g006550	Auxin influx carrier (AUX1 LAX family)	Auxin transporter-like protein 2	-1.560
Cg4g002180	Auxin-responsive protein IAA	Auxin-responsive protein IAA29	3.122
Cg4g006940	Auxin-responsive protein IAA	Auxin-responsive protein IAA29	2.241
Cg4g021810	Auxin-responsive protein IAA	Auxin-responsive protein IAA33	1.792
Cg5g036600	Auxin response factor	Auxin response factor 7	9.405
CgUng005390	SAUR family protein	Auxin-responsive protein SAUR24 {ECO:0000305}	-1.813
	Cytokinin		
Cg1g013900	Histidine-containing phosphotransfer peotein	Histidine-containing phosphotransfer protein 4	6.555
Cg8g005300	Two-component response regulator ARR-B family	Myb family transcription factor EFM {ECO:0000303丨PubMed:25132385}	-1.061
	Gibberellin		
Cg2g032630	Phytochrome-interacting factor 3	Transcription factor bHLH149	1.435
Cg6g013650	Gibberellin receptor GID1 [EC:3.-.-.-]	Probable carboxylesterase 15	1.352
Cg3g006980	Gibberellin receptor GID1 [EC:3.-.-.-]	Probable carboxylesterase 6	2.007
Cg5g009820	DELLA protein	Scarecrow-like protein 28	2.092
Cg1g006390	DELLA protein	Scarecrow-like protein 32	4.915
Cg9g001400	DELLA protein	DELLA protein RGL2	-5.213
Cg9g001430	DELLA protein	DELLA protein RGL2	3.503
	Abscisic acid		
Cg6g015730	ABA responsive element binding factor	Protein ABSCISIC ACID-INSENSITIVE 5	3.881
Cg5g001390	Protein phosphatase 2C [EC:3.1.3.16]	Probable protein phosphatase 2C 8	3.181
Cg7g000680	Protein phosphatase 2C [EC:3.1.3.16]	Protein phosphatase 2C 37	1.252
Cg8g023080	Protein phosphatase 2C [EC:3.1.3.16]	Protein phosphatase 2C 3	2.730
Cg9g028610	Protein phosphatase 2C [EC:3.1.3.16]	Probable protein phosphatase 2C 8	1.958
CgUng003480	Protein phosphatase 2C [EC:3.1.3.16]	Protein phosphatase 2C 56	1.448
Cg6g009750	Abscisic acid receptor PYR/PYL family	Abscisic acid receptor PYL4	-2.059
Cg7g002520	Abscisic acid receptor PYR/PYL family	Abscisic acid receptor PYL4	-2.222
	Ethylene		
Cg2g038570	Mitogen-activated protein kinase kinase 4/5 [EC:2.7.12.2]	Protein LIGHT-DEPENDENT SHORT HYPOCOTYLS 6	1.067
Cg9g008980	Ethylene-insensitive protein 3	Phosphate transporter PHO1	1.909
Cg4g007230	EIN3-binding F-box protein	EIN3-binding F-box protein 1	-1.226
	Brassinosteroid		
Cg3g020940	Cyclin D3, plant	Cyclin-D3-1	2.052
Cg2g043490	Protein brassinosteroid insensitive 1 [EC:2.7.10.1 2.7.11.1]	Calmodulin-binding receptor kinase CaMRLK {ECO:0000305}	1.303
Cg1g026820	Protein brassinosteroid insensitive 1 [EC:2.7.10.1 2.7.11.1]	Protein BRASSINOSTEROID INSENSITIVE 1 {ECO:0000303丨PubMed:9298904}	-1.285
Cg3g021510	BRI1 kinase inhibitor 1	BRI1 kinase inhibitor 1	-1.439
	Jasmonic acid		
Cg1g007240	Transcription factor MYC2	Transcription factor bHLH25	8.612
Cg2g040740	Transcription factor MYC2	Transcription factor ILR3	-1.273

		Salicylic acid	
Cg8g014140	Transcription factor TGA	Transcription factor TGA9 {ECO:0000303|PubMed:20805327}	1.538
Cg1g008410	Pathogenesis-related protein 1	Basic form of pathogenesis-related protein 1	7.160
Cg7g012290	Regulatory protein NPR1	BTB/POZ domain and ankyrin repeat-containing protein NPR2 {ECO:0000305}	-1.389
Cg7g012320	Regulatory protein NPR1	BTB/POZ domain and ankyrin repeat-containing protein NPR2 {ECO:0000305}	-2.602

Table S16. DEGs involved in hormone activated and/or mediated signaling pathways in leaves of *C. grandis* seedlings exposed to Cu-toxicity

Accession No.	KEGG	Swiss-Prot	Log_2(FC)
	Auxin-activated signaling pathway (GO:0009734)		
Cg1g000490	Uncharacterized protein	Protein PIN-LIKES 5 {ECO:0000303丨PubMed:22504182}	1.367
Cg1g010480		Auxin-binding protein ABP19a	-1.866
Cg1g012930	ATP-binding cassette, subfamily B (MDR/TAP), member 1 [EC:3.6.3.44]	ABC transporter B family member 19	-2.700
Cg6g024720	ATP-binding cassette, subfamily B (MDR/TAP), member 1 [EC:3.6.3.44]	ABC transporter B family member 19	1.122
Cg1g021380		Probable N-acetyltransferase HLS1	1.638
Cg3g017880	Auxin efflux carrier family	Auxin efflux carrier component 6 {ECO:0000303丨PubMed:15817418}	7.737
Cg6g017830	Auxin efflux carrier family	Probable auxin efflux carrier component 1c {ECO:0000305}	2.613
Cg3g023440	Integrin-linked kinase-associated serine/threonine phosphatase 2C [EC:3.1.3.16]	Protein WALLS ARE THIN 1	2.919
Cg4g002180	Auxin-responsive protein IAA	Auxin-responsive protein IAA29	3.122
Cg4g006940	Auxin-responsive protein IAA	Auxin-responsive protein IAA29	2.241
Cg4g021810	Auxin-responsive protein IAA	Auxin-responsive protein IAA33	1.792
Cg5g036600	Auxin response factor	Auxin response factor 7	9.405
Cg6g006550	Auxin influx carrier (AUX1 LAX family)	Auxin transporter-like protein 2	-1.560
CgUng005390	SAUR family protein	Auxin-responsive protein SAUR24 {ECO:0000305}	-1.813
Cg4g018940		Protein BIG GRAIN 1-like B {ECO:0000305}	1.824
Cg5g006140	E1A/CREB-binding protein [EC:2.3.1.48]	BTB/POZ and TAZ domain-containing protein 2	-1.821
Cg5g016500	Protein-serine/threonine kinase [EC:2.7.11.-]	Serine/threonine-protein kinase WAG1	-1.725
Cg6g009060		Auxin-induced in root cultures protein 12	-1.012
Cg7g016040		Protein BIG GRAIN 1-like E {ECO:0000305}	-2.521
	Gibberellic acid mediated signaling pathway (GO:0009740)		
Cg3g022490	Beta-galactosidase [EC:3.2.1.23]	Protein indeterminate-domain 1 {ECO:0000303丨PubMed:16784536	1.026
Cg4g022720		Transcription factor IBH1-like 1 {ECO:0000303丨PubMed:24505057}	-2.837
Cg7g019960	Xyloglucan glycosyltransferase 4 [EC:2.4.1.-]	Gibberellin-regulated protein 14	5.707
Cg8g005300	Two-component response regulator ARR-B family	Myb family transcription factor EFM {ECO:0000303丨PubMed:25132385}	-1.061
Cg8g023080	Protein phosphatase 2C [EC:3.1.3.16]	Protein phosphatase 2C 3	2.730
Cg9g001400	DELLA protein	DELLA protein RGL2	-5.213
Cg9g001430	DELLA protein	DELLA protein RGL2	3.503
	Ethylene-activated signaling pathway (GO:0009873)		
Cg1g013070	Acetylajmaline esterase [EC:3.1.1.80]	GDSL esterase/lipase 1 {ECO:0000303丨PubMed:16126835}	1.365

Cg3g017880	Auxin efflux carrier family	Auxin efflux carrier component 6 {ECO:0000303|PubMed:15817418}	7.737
Cg5g001740	Pathogenesis-related genes transcriptional activator PTI6	Ethylene-responsive transcription factor CRF2	1.867
Cg1g019920	EREBP-like factor	Ethylene-responsive transcription factor ERF003	2.059
Cg3g005210		Ethylene-responsive transcription factor WRI1	2.575
Cg4g017840	EREBP-like factor	Ethylene-responsive transcription factor ERF016	1.591
Cg4g004100	EREBP-like factor	Dehydration-responsive element-binding protein 3	1.644
Cg4g015400	EREBP-like factor	Ethylene-responsive transcription factor ERF035	-1.807
Cg4g001690	EREBP-like factor	Ethylene-responsive transcription factor ERF003	-2.375
Cg5g037790	EREBP-like factor	Ethylene-responsive transcription factor RAP2-3	-3.275
Cg5g044100	EREBP-like factor	Ethylene-responsive transcription factor ERF034	-1.565
Cg5g044340	EREBP-like factor	Ethylene-responsive transcription factor 13	-1.305
Cg7g014820	RAV-like factor	AP2/ERF and B3 domain-containing transcription repressor RAV2	-2.294
Cg9g022880	EREBP-like factor	Ethylene-responsive transcription factor ERF106	-1.269
Cg4g007230	EIN3-binding F-box protein	EIN3-binding F-box protein 1	-1.226
Brassinosteroid mediated signaling pathway (GO:0009742)			
Cg1g026820	Protein brassinosteroid insensitive 1 [EC:2.7.10.1 2.7.11.1]	Protein BRASSINOSTEROID INSENSITIVE 1 {ECO:0000303|PubMed:9298904}	-1.285
Cg1g028040	Somatic embryogenesis receptor kinase 1 [EC:2.7.10.1 2.7.11.1]	LRR receptor kinase BAK1 {ECO:0000305}	-2.102
Cg3g021510	BRI1 kinase inhibitor 1	BRI1 kinase inhibitor 1	-1.439
Cg4g022720		Transcription factor IBH1-like 1 {ECO:0000303|PubMed:24505057}	-2.837
Cg5g009820	DELLA protein	Scarecrow-like protein 28	2.092
Cg9g005160		Probable membrane-associated kinase regulator 1	1.083
Abscisic acid-activated signaling pathway (GO:0009738)			
Cg1g001510	Oomolarity two-component system, sensor histidine kinase NIK1 [EC:2.7.13.3]	Histidine kinase 1	3.571
Cg1g007430	AP-1 complex subunit beta-1	Glycine-rich domain-containing protein 1 {ECO:0000303|Ref.4}	-1.659
Cg1g012650	Ras-related C3 botulinum toxin substrate 1	Ras-related C3 botulinum toxin substrate 1	2.152
Cg2g041170	Potein FLOWERING LOCUS T	Protein MOTHER of FT and TFL1	2.386
Cg3g013260	Hmeobox-leucine zipper protein	Homeobox-leucine zipper protein HAT22	-2.487
Cg3g020400	E3 ubiquitin-protein ligase RNF181 [EC:2.3.2.27]	E3 ubiquitin-protein ligase AIP2	6.069
Cg4g014320	AarF domain-containing kinase	Transcription factor SRM1 {ECO:0000305}	1.398
Cg5g006140	E1A/CREB-binding protein [EC:2.3.1.48]	BTB/POZ and TAZ domain-containing protein 2	-1.821
Cg5g035910		EID1-like F-box protein 3	2.693
Cg6g009750	Abscisic acid receptor PYR/PYL family	Abscisic acid receptor PYL4	-2.059
Cg6g015730	ABA responsive element binding factor	Protein ABSCISIC ACID-INSENSITIVE 5	3.881
Cg7g000680	Protein phosphatase 2C [EC:3.1.3.16]	Protein phosphatase 2C 37	1.252
Cg7g002520	Abscisic acid receptor PYR/PYL family	Abscisic acid receptor PYL4	-2.222

Cg8g023080	Protein phosphatase 2C [EC:3.1.3.16]	Protein phosphatase 2C 3	2.730
Cg9g026770		Low-temperature-induced 65 kDa protein	1.431
CgUng002190	AarF domain-containing kinase	Transcription factor SRM1 {ECO:0000305}	-1.326
CgUng003480	Protein phosphatase 2C [EC:3.1.3.16]	Protein phosphatase 2C 56	1.448
Salicylic acid mediated signaling pathway (GO:0009863)			
Cg2g042710	Glutaredoxin 3	Glutaredoxin-C9	3.564
Cg7g006930	(R)-mandelonitrile lyase [EC:4.1.2.10]	Salicylic acid-binding protein 2	1.429
Cg9g029620	YTH domain-containing family protein	30-kDa cleavage and polyadenylation specificity factor 30 {ECO:0000303|PubMed:18479511}	1.074
Cg8g007780	Mitochondrial chaperone BCS1	Protein HYPER-SENSITIVITY-RELATED 4	-2.370
Cytokinin-activated signaling pathway (GO:0009736)			
Cg1g013900	Histidine-containing phosphotransfer peotein	Histidine-containing phosphotransfer protein 4	6.555
Cg5g001740	Pathogenesis-related genes transcriptional activator PTI6	Ethylene-responsive transcription factor CRF2	1.867
Jasmonic acid mediated signaling pathway (GO:0009867)			
Cg2g042710	Glutaredoxin 3	Glutaredoxin-C9	3.564

Table S17. DEGs involved in hormone metabolism in leaves of *C. grandis* seedlings exposed to Cu-toxicity

Accession No.	KEGG	Swiss-Prot	$Log_2(FC)$
Abscisic acid metabolic process (GO:0009687)			
Cg1g001690	Cytochrome P450 family 26 subfamily A	Abscisic acid 8'-hydroxylase 1	6.219
Cg4g014320	AarF domain-containing kinase	Transcription factor SRM1 {ECO:0000305}	1.398
CgUng002190	AarF domain-containing kinase	Transcription factor SRM1 {ECO:0000305}	-1.326
Cg2g044950	9-cis-epoxycarotenoid dioxygenase [EC:1.13.11.51]	9-cis-epoxycarotenoid dioxygenase NCED1, chloroplastic	-3.481
Cg4g002980	Zeaxanthin epoxidase [EC:1.14.15.21]	Zeaxanthin epoxidase, chloroplastic	-1.171
Abscisic acid biosynthetic process (GO:0009688)			
Cg2g044950	9-cis-epoxycarotenoid dioxygenase [EC:1.13.11.51]	9-cis-epoxycarotenoid dioxygenase NCED1, chloroplastic	-3.481
Cg4g002980	Zeaxanthin epoxidase [EC:1.14.15.21]	Zeaxanthin epoxidase, chloroplastic	-1.171
CgUng002190	AarF domain-containing kinase	Transcription factor SRM1 {ECO:0000305}	-1.326
Cg4g014320	AarF domain-containing kinase	Transcription factor SRM1 {ECO:0000305}	1.398
Cytokinin metabolic process (GO:0009690)			
Cg3g023850	Uncharacterized protein	Cytokinin riboside 5'-monophosphate phosphoribohydrolase LOG7	2.170
Cg5g004060	Cytokinin dehydrogenase [EC:1.5.99.12]	Cytokinin dehydrogenase 1	1.246
Cg7g022650	Cytokinin dehydrogenase [EC:1.5.99.12]	Cytokinin dehydrogenase 6	-1.092
Cg4g018790	Cytokinin dehydrogenase [EC:1.5.99.12]	Cytokinin dehydrogenase 7	-2.115
Cytokinin biosynthetic process (GO:0009691)			
Cg3g023850	Uncharacterized protein	Cytokinin riboside 5'-monophosphate phosphoribohydrolase LOG7	2.170
Auxin metabolic process (GO:0009850)			
Cg1g003960	Indole-3-pyruvate monooxygenase [EC:1.14.13.168]	Indole-3-pyruvate monooxygenase YUCCA2	2.300
Cg2g013180	Pathogen-inducible salicylic acid glucosyltransferase [EC:2.4.1.-]	UDP-glycosyltransferase 74E2	2.959
Cg3g023440	Integrin-linked kinase-associated serine/threonine phosphatase 2C [EC:3.1.3.16]	Protein WALLS ARE THIN 1	2.919
Cg5g030310	Cytochrome P450 family 71 subfamily A	Cytochrome P450 83B1	3.608
Cg7g004010	Pheophorbidase [EC:3.1.1.82]	Methylesterase 17	2.698
Cg8g024170	Phosphatidylinositol phospholipase C, delta [EC:3.1.4.11]	Phosphoinositide phospholipase C 2	2.238
Cg4g021380	Gibberellin 2-oxidase [EC:1.14.11.13]	2-oxoglutarate-dependent dioxygenase DAO	-1.022
Cg7g001270	Anthranilate synthase component I [EC:4.1.3.27]	Anthranilate synthase alpha subunit 1, chloroplastic	-1.583
Auxin biosynthetic process (GO:0009851)			
Cg1g003960	Indole-3-pyruvate monooxygenase [EC:1.14.13.168]	Indole-3-pyruvate monooxygenase YUCCA2	2.300
Cg3g023440	Integrin-linked kinase-associated serine/threonine phosphatase 2C [EC:3.1.3.16]	Protein WALLS ARE THIN 1	2.919
Cg5g030310	Cytochrome P450 family 71 subfamily A	Cytochrome P450 83B1	3.608
Cg8g024170	Phosphatidylinositol phospholipase C,	Phosphoinositide phospholipase C 2	2.238

	delta [EC:3.1.4.11]		
Cg7g001270	Anthranilate synthase component I [EC:4.1.3.27]	Anthranilate synthase alpha subunit 1, chloroplastic	-1.583
Auxin transport (GO:0060918)			
Cg7g021600	Golgi-specific brefeldin A-resistance guanine nucleotide exchange factor 1	ARF guanine-nucleotide exchange factor GNOM	1.069
Cg6g017830	Auxin efflux carrier family	Probable auxin efflux carrier component 1c {ECO:0000305}	2.613
Cg3g017880	Auxin efflux carrier family	Auxin efflux carrier component 6 {ECO:0000303|PubMed:15817418}	7.737
Cg3g023440	Integrin-linked kinase-associated serine/threonine phosphatase 2C [EC:3.1.3.16]	Protein WALLS ARE THIN 1	2.919
Cg4g018940		Protein BIG GRAIN 1-like B {ECO:0000305}	1.824
Cg4g024570	5'-AMP-activated protein kinase, catalytic alpha subunit [EC:2.7.11.11]	CBL-interacting serine/threonine-protein kinase 6	1.132
Cg6g024720	ATP-binding cassette, subfamily B (MDR/TAP), member 1 [EC:3.6.3.44]	ABC transporter B family member 19	1.122
Cg1g012930	ATP-binding cassette, subfamily B (MDR/TAP), member 1 [EC:3.6.3.44]	ABC transporter B family member 19	-2.700
Cg7g016040		Protein BIG GRAIN 1-like E {ECO:0000305}	-2.521
Cg5g016500	Protein-serine/threonine kinase [EC:2.7.11.-]	Serine/threonine-protein kinase WAG1	-1.725
Brassinosteroid biosynthetic process (GO:0016132)			
Cg1g001690	Cytochrome P450 family 26 subfamily A	Abscisic acid 8'-hydroxylase 1	6.219
Cg1g009640	3-epi-6-deoxocathasterone 23-monooxygenase [EC:1.14.13.112]	3-epi-6-deoxocathasterone 23-monooxygenase	-1.351
Cg3g021510	BRI1 kinase inhibitor 1	BRI1 kinase inhibitor 1	-1.439
Salicylic acid metabolic process (GO:0009696)			
Cg5g021790	Saccharopepsin [EC:3.4.23.25]	Aspartic proteinase CDR1	-4.185
Cg5g021820	Saccharopepsin [EC:3.4.23.25]	Aspartic proteinase CDR1	-1.398
Cg1g017890	2'-deoxymugineic-acid 2'-dioxygenase / mugineic-acid 3-dioxygenase [EC:1.14.11.24 1.14.11.25]	Protein DMR6-LIKE OXYGENASE 2 {ECO:0000303|PubMed:25376907}	8.980
Cg1g017900	2'-deoxymugineic-acid 2'-dioxygenase / mugineic-acid 3-dioxygenase [EC:1.14.11.24 1.14.11.25]	Protein DMR6-LIKE OXYGENASE 2 {ECO:0000303|PubMed:25376907}	6.975
Salicylic acid catabolic process (GO:0046244)			
Cg1g017890	2'-deoxymugineic-acid 2'-dioxygenase / mugineic-acid 3-dioxygenase [EC:1.14.11.24 1.14.11.25]	Protein DMR6-LIKE OXYGENASE 2 {ECO:0000303|PubMed:25376907}	8.980
Cg1g017900	2'-deoxymugineic-acid 2'-dioxygenase / mugineic-acid 3-dioxygenase [EC:1.14.11.24 1.14.11.25]	Protein DMR6-LIKE OXYGENASE 2 {ECO:0000303|PubMed:25376907}	6.975
Jasmonic acid metabolic process (GO:0009694)			
Cg2g037340	E3 ubiquitin-protein ligase ATL10/75/76/77/78 [EC:2.3.2.27]	RING-H2 finger protein ATL73	-2.282
Cg7g007580	OPC-8:0 CoA ligase 1 [EC:6.2.1.-]	4-coumarate--CoA ligase-like 5	5.062
Cg3g016650	Jasmonoyl-L-amino acid hydrolase	IAA-amino acid hydrolase ILR1-like 6	1.585

	[EC:3.5.1.127]	{ECO:0000303|PubMed:11923288}	
Jasmonic acid biosynthetic process (GO:0009695)			
Cg2g037340	E3 ubiquitin-protein ligase ATL10/75/76/77/78 [EC:2.3.2.27]	RING-H2 finger protein ATL73	-2.282
Cg7g007580	OPC-8:0 CoA ligase 1 [EC:6.2.1.-]	4-coumarate--CoA ligase-like 5	5.062
Gibberellin biosynthetic process (GO:0009686)			
Cg2g023660	Gibberellin 2-oxidase [EC:1.14.11.13]	Gibberellin 2-beta-dioxygenase 8	3.126
Cg2g044860	Gibberellin 2-oxidase [EC:1.14.11.13]	Gibberellin 2-beta-dioxygenase	2.853

Table S18. Specific primer pairs used for qRT-PCR expression analysis

Genes	Accession No.	Forward primers (5′→3′)	Reverse primers (5′→3′)
Vicilin-like seed storage protein At2g18540	Cg1g003870	ACTTGACAAAGGGCTCG	CTGTGCTCGGACAAACC
LanC-like protein GCL1	Cg1g008400	TAGAGGAGGGATTTACGC	CCCGAGATGCTTGTTTA
Wall-associated receptor kinase-like 10	Cg1g015970	TGGTATTAGCAGGGATT	ATGATTCGGTGAAAGAT
NAC domain-containing protein 72	Cg2g021360	TTCAGCCCCAGGGATAG	CAACCTTACGACCTTCAGT
Bidirectional sugar transporter SWEET16	Cg2g031810	AATGCCGTAGGGTTCGT	TGTGGGCGGATCTTTCT
Protein CHUP1, chloroplastic	Cg2g038560	TCAAACGACTGCTACAA	ACTCTGACAACTCTGCTT
Protein MOTHER of FT and TFL1	Cg2g041170	TCAGAAAGCGTTGAGATTG	TCAGCAGAAGCAGAGGG
Plant cysteine oxidase 1 {ECO:0000303|PubMed:24599061}	Cg5g031660	TTTCTTCCTCCAACTGC	ACTGTCGGCTATCAACTT
Hypothetical protein CICLE_v10033235mg [Citrus clementina]	Cg7g022450	GATTTGCGATGCTTGCT	GGTTCTTGTCACCTTTTC
ATP-dependent zinc metalloprotease FTSH 6, chloroplastic	Cg6g024950	AAGCAGTGATGTCGTGA	TTCCCTGTTATTCCTTATGTG
Pectinesterase 3	Cg1g013250	GAGTGAAATGGAAGGGAT	CAAACCAAGGGAGAATG
Lectin	Cg4g006720	AAAAGTGGACGAGCAAT	TCTGGCAGGTAAATGTG
Probable calcium-binding protein CML41	Cg1g003570	AAACTCCTCCTTGTCTGC	CTTGAAAATCCAATAAACTG
LOB domain-containing protein 39	Cg3g012700	CGCCCGCTCAGTCAAAT	GCAAGCACGACCTCAGTAT
B-box domain protein 31 {ECO:0000303|PubMed:19920209}	Cg2g041050	TGATGACGATGATGGGG	GATTCTTGCTTTGTTGAG
Gamma-glutamyl peptidase 5 {ECO:0000305}	Cg4g023690	TTCGGGGAAGAAGGAGA	TAAGGGCTACCGCTGAT
36.4 kDa proline-rich protein	Cg6g012200	GATTCTCCTCTTGAACTTGGGTA	GGGGTGGCTTGTGATGC
Probable alpha-mannosidase At5g13980 {ECO:0000305}	Cg7g011170	GATTCCAGTGGTCGTGA	TTACTTCCATCGTCTCC
Cyclin-D3-1	Cg3g020940	TCCTCCTACAGGGTGCC	TTCAATAGTTGCTTCTCATA
Monothiol glutaredoxin-S2	Cg9g003340	ATGGCAATGGAGCGAGTA	CACCGCCGATGAACACG
Endochitinase	Cg8g000800	TGAAGGCTGCCAAAGTA	ACCAAAGGCTGGAAAAG
Actin	Cg1g026080	AGAACTATGAACTGCCTGATGGC	GCTTGGAGCAAGTGCTGTGATT
U4/U6 small nuclear ribonucleoprotein PRP31 (PRPF31)	Cg7g019550	ACTCATGGGAACGGCTGGTGGTC	TCGGCAGGCACGCATCCTTAGAG
